# *In silico* design of novel multi-epitope peptide vaccine against *Neospora caninum* induced cattle abortion targeting extracellular GRA2 and Nc-p43 protein

**DOI:** 10.1038/s41598-025-26139-1

**Published:** 2025-11-26

**Authors:** Md. Nafij Mashrur, Md. Imranuzzaman, Hridoy Barua, Md. Rakibul Hasan, Faria Khan Hridy, Tasnim Sayem Sakib, Nurul Amin Rani, Mostafizor Rahman, Golam Ahsan, Tanvir Ashraf, Sofiur Muktadir, Anika Binte Belal, Apurba Roy, A. S. M. Mohiuddin, Tushar Ahmed Shishir, Hemayet Hossain, Md. Masudur Rahman, Mst Rubaiat Nazneen Akhand, Md. Mahfujur Rahman

**Affiliations:** 1 Department of Biochemistry and Molecular Biology, Gono University, Dhaka, 1344 Bangladesh; 2 Department of Agriculture and Environmental Sciences, Lincoln University, Jefferson City, MO 65101 USA; 3 Faculty of Biotechnology and Genetic Engineering, Sylhet Agricultural University, Sylhet, 3100 Bangladesh; 4 Department of Animal and Fish Biotechnology, Sylhet Agricultural University, Sylhet, 3100 Bangladesh; 5 Department of Dairy & Poultry Science, Hajee Mohammad Danesh Science & Technology University, Dinajpur, 5200 Bangladesh; 6 Faculty of Veterinary, Animal and Biomedical Sciences, Sylhet Agricultural University, Sylhet, 3100 Bangladesh; 7 Department of Microbiology, Hajee Mohammad Danesh Science & Technology University, Dinajpur, 5200 Bangladesh; 8 Faculty of Animal Science and Veterinary Medicine, Patuakhali Science and Technology University, Dumki, 8602 Patuakhali Bangladesh; 9 Department of Medicine and Surgery, Chattogram Veterinary and Animal Sciences University, Chattogram, 4202 Bangladesh; 10 Department of Animal Production and Management, Sher-e-Bangla Agriculture University, Sher-e-Bangla Nagar, Dhaka, 1207 Bangladesh; 11 Department of Pathology, Sylhet Agricultural University, Sylhet, 3100 Bangladesh; 12 Department of Mathematics and Natural Sciences, BRAC University, Dhaka, 1212 Bangladesh; 13 Department of Anatomy and Histology, Sylhet Agricultural University, Sylhet, 3100 Bangladesh; 14 Department of Biochemistry and Chemistry, Sylhet Agricultural University, Sylhet, 3100 Bangladesh; 15 Department of Medicine, Sylhet Agricultural University, Sylhet, 3100 Bangladesh

**Keywords:** Neospora caninum, Multi-epitope peptide (MEP) vaccine, Molecular docking, Molecular dynamics simulation, *In Silico* study, Biotechnology, Computational biology and bioinformatics, Immunology, Microbiology

## Abstract

*Neospora caninum* (NC) is a protozoan infection causing neosporosis, a disease that leads to substantial economic loss in livestock, especially in cattle, sheep, and goats. Unfortunately, there is presently no viable vaccination or authorized therapy available. Our research applied a bioinformatics approach to design a multi-epitope peptide (MEP) vaccine aimed at the pathogenic proteins GRA2 and Nc-p43 of NC, which are critical in parasite-mediated antigenicity and host interactions. Consequently, our research employs an in-silico methodology, including protein sequence retrieval, epitope prediction, vaccine design, structural analysis, molecular docking, molecular dynamics simulations, immunological simulation, and codon optimization using in silico cloning. We conducted analyses of antigenicity, allergenicity, toxicity, topology, and immunogenicity using multiple bioinformatics methods and identified 4 CTL, 4 HTL, and 2 B-cell epitopes. The vaccine design was created by integrating an adjuvant and a PADRE sequence to enhance immunogenicity, along with linkers (AAY, GPGPG, KK) to facilitate appropriate structural assembly, which was then analyzed for optimal complete profiles. Molecular docking with the *Bos taurus* (cattle) TLR9 receptor demonstrated a robust binding affinity, scoring − 1183.4, while molecular dynamics (MD) simulations over 50 ns validated persistent interactions between the vaccine and immune receptors. Moreover, immunological models forecasted a robust adaptive immune response, marked by an increase in the production of cytokines and the establishment of memory T-lymphocytes. Ultimately, codon optimization and in silico cloning validated the capacity for effective expression in *E. coli*. The results demonstrate that the developed vaccine has considerable immunogenic potential against NC. Nonetheless, more in vitro and in vivo studies are necessary to confirm its efficiency.

**Supplementary Information:**

The online version contains supplementary material available at 10.1038/s41598-025-26139-1.

## Introduction

*Neospora caninum* (NC) is an obligate intracellular protozoan of the family Sarcocystidae (phylum Apicomplexa) that causes neosporosis, leading to bovine abortion and reproductive issues in cattle, and paralysis in dogs^1,2^. The organism was initially identified in dogs in 1984, and it was subsequently designated as a novel genus and species in 1988 ^3^. NC’s erratic behavior and propensity for dissemination render it a substantial threat, particularly to immunocompromised animals. In the Western Amazon region of Brazil, NC was detected in 60.6% of the sheep^4^. Globally, seroprevalence in livestock varies widely, with reports ranging from 10% to over 60%, highlighting the widespread impact of the parasite.

NC has a complex life cycle with both horizontal and vertical transmission involving predators and herbivores. It exists in three stages: tachyzoites, bradyzoites, and oocysts. Infected canids shed oocysts in feces, contaminating food, water, or the environment. Pregnant cows and dogs can transmit NC to offspring via the placenta, leading to congenital infection, abortion, stillbirth, or chronically infected calves^5^. This stage, which multiplies quickly, infiltrates different host cells and causes illness. Both definitive and intermediate hosts’ tissues contain tachyzoites^6^. Within Bradyzoites, these are slow-replicating types that mostly affect intermediate hosts like cattle’s central nervous systems, where they create tissue cysts. During pregnancy, they may reactivate and cause transplacental transmission^1^. Such mechanisms of persistence and reactivation not only complicate disease control but also create major challenges for vaccine development.

In Bangladesh, this parasite was found in 16% of aborted cattle fetuses, indicating a significant association with pregnancy stages and the presence of dogs^7^. Similarly, in the Kurdistan area of Iraq, it was found in 20.3% of aborted dams, with older age and dog exposure emerging as important risk factors^8^.

NC pathogenicity is mostly ascribed to important virulence-associated proteins, such as dense granule proteins (GRAs) and surface antigens (SAGs). Among these, dense granule protein 2 (GRA2) and surface protein Nc-p43 play critical roles in host–parasite interactions and significantly contribute to disease severity in cattle and dogs^9^. GRA2 facilitates the formation of the intravacuolar network within the parasitophorous vacuole, enabling efficient nutrient uptake from the host^10^. In contrast, Nc-p43 is involved in host-cell adhesion and invasion, and its differential expression in tachyzoites and bradyzoites supports stage-specific immune evasion^10^.

Because of their strong immunogenicity and role in parasite survival, these proteins are considered promising vaccine targets.

Immunoinformatics has shown efficacy in identifying antigenic peptides of T-cell and B-cell epitopes for the construction of vaccines using in silico vaccine design methods^11–13^. In comparison to the previously proposed epitope-based vaccination that targeted the NC proteins, the antigenicity, stability, and other features of the created vaccine were also tested^13^. Because of their high immunogenicity, GRA2 and Nc-p43 have been extensively researched as possible vaccine candidates.

Till now, several vaccination approaches have been developed to prevent NC infection, such as the use of recombinant subunit vaccines^14^, live or inactivated attenuated vaccines^15^, recombinant parasite antigens, and DNA vaccines^16^. Furthermore, neosporosis had a significant economic influence, particularly concerning the ruminant sector^2^. In these circumstances, vaccination with MEP vaccines, including several T lymphocyte and B-cell epitopes from key antigens targeting different diseases, has led to a markedly enhanced level of humoral and cellular immune responses, as well as an extended lifespan^13^.

Therefore, this study aimed to design and evaluate a novel in silico multi-epitope peptide vaccine construct against NC by targeting GRA2 and Nc-p43 proteins. Using advanced immunoinformatics, structural modeling, molecular docking, and dynamics simulations, we sought to predict vaccine stability, immunogenicity, and potential immune responses. This work provides foundational insights that may accelerate experimental validation and future vaccine development against neosporosis.

## Methods

**Fig. 1:**
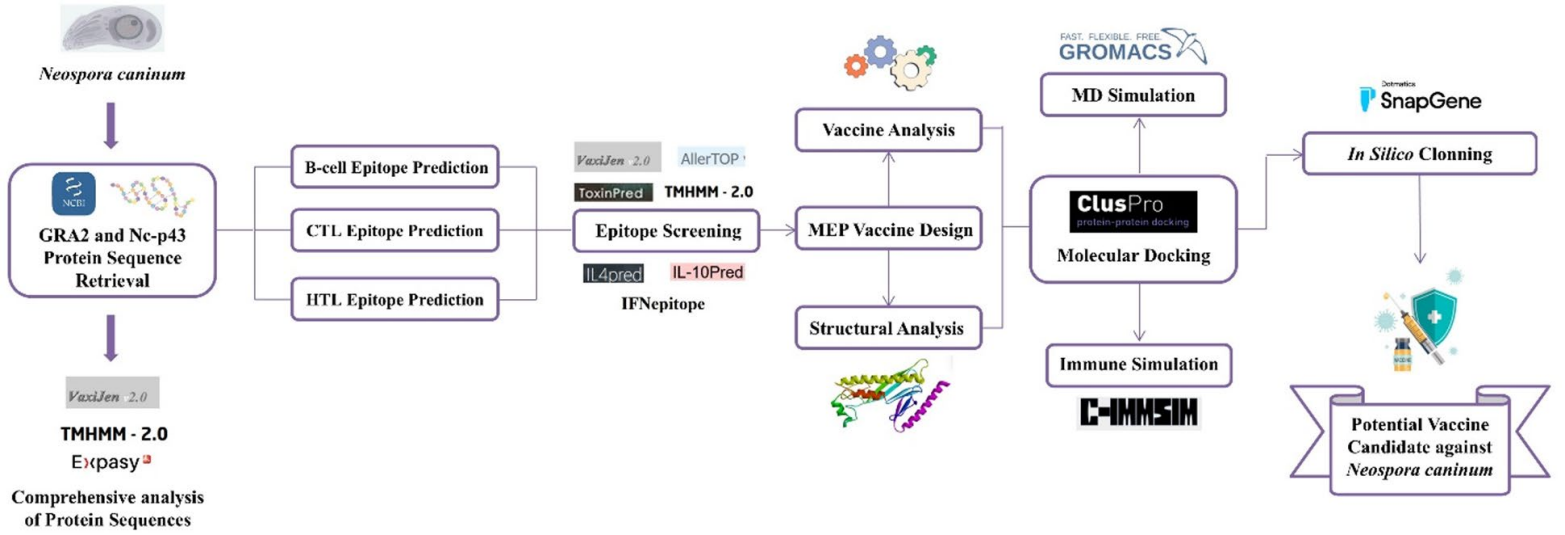
The overall workflow of this study.

Protein sequences of GRA2 and Nc-p43 from Neospora caninum were retrieved and analyzed through immunoinformatics tools to predict CTL, HTL, and B-cell epitopes, which were then assembled into a MEP vaccine construct using suitable linkers and adjuvants. The construct was subsequently evaluated through structural modeling, molecular docking with TLR9, molecular dynamics simulations, immune response simulations, and codon optimization to assess its stability, immunogenicity, and expression potential. An illustration of the entire methodology is provided in Fig. 1.

### Protein sequence collection and comprehensive analysis

The selected complete two protein sequences, dense granule protein 2 (accession number: AAC39122.1), and surface protein Nc-p43 (accession number: AAC15250.1), of the chosen parasite *NC*, were retrieved by searching the National Center for Biotechnology Information (NCBI) database (https://www.ncbi.nlm.nih.gov/) ^17^ in FASTA file format. Subsequently, the antigenicity and protein topology of the two selected proteins were examined using VaxiJen v2.0 (http://www.ddgpharmfac.net/vaxijen/) ^18^ and TMHMM online server v2.0 (http://www.cbs.dtu.dk/services/TMHMM/) ^19^. Additionally, the physicochemical properties of the two chosen proteins were determined using the ProtParam ExPASy tool (https://web.expasy.org/protparam/) ^20^.

### B-cell epitope prediction

The primary aim of predicting B-cell epitopes is to identify probable antigens that interact with B cells and provoke an immune response. The B-cell epitope of the chosen protein was forecasted utilizing IEDB v2.28 (http://tools.iedb.org/bcell/) ^21,22^. Alongside several algorithms, including the Emini surface accessibility prediction, Bepipred linear epitope prediction analysis, Kolaskar and Tongaonkar antigenicity scale, Karplus and Schulz flexibility prediction, Chou and Fasman beta turn prediction, and Parker hydrophilicity assessment.

### B-cell epitope screening

The VaxiJen v2.0 (https://www.ddg-pharmfac.net/vaxijen/VaxiJen/VaxiJen.html) web server was used to evaluate the antigenicity level at the threshold (> 0.4). To evaluate the allergy susceptibility analysis, there are four online servers were used: AllerTOP v2.0 (https://www.ddg-pharmfac.net/AllerTOP/) ^23^, AllergenOnline v1.0 (http://www.allergenonline.org/databasefasta.shtml)^24^, Allermatch v1.0 (http://www.allermatch.org/allermatchsearch/form)^25^, and AllergenFP v1.0 (https://ddg-pharmfac.net/AllergenFP/) ^12^. The ToxinPred2 online service (http://crdd.osdd.net/raghava/toxinpred/) was applied to assess the toxicity level of epitopes^26^. Lastly, the TMHMM v2.0 (https://services.healthtech.dtu.dk/services/TMHMM-2.0/) method was employed to investigate the topology of the epitopes^19^.

### T cell epitope prediction

CTL (Cytotoxic T-lymphocytes) play a vital role in MHC-I mediated reactions by targeting and eliminating host cells infected with NC. MHC II plays a vital role in triggering HTL (Helper T-lymphocytes), which are crucial for adaptive immunity. The server NetMHCpan v4.1 (https://services.healthtech.dtu.dk/services/NetMHCpan-4.1/) was utilized to forecast MHC class I epitopes using all available bovine leukocyte antigen (BoLA) alleles, while the NetMHCIIpan v4.1 server (https://services.healthtech.dtu.dk/services/NetMHCIIpan-4.1/) was employed to screen the protein epitopes for MHC II epitopes using BoLA class-II alleles^27,28^.

### T cell epitopes comprehensive analysis: antigenicity, allergenicity, transmembrane topology, toxicity, and immunogenicity include IL4, IL10, and IFN-γ

As well as the comprehensive analysis of B-cell epitopes, the antigenic level of epitopes was evaluated using the VaxiJen v2.0 (https://www.ddg-pharmfac.net/vaxijen/VaxiJen/VaxiJen.html) online server. The AllerTOP v2.0 (https://www.ddg-pharmfac.net/AllerTOP/) ^23^, AllergenOnline v1.0 (http://www.allergenonline.org/databasefasta.shtml)^24^, Allermatch v1.0 (http://www.allermatch.org/allermatchsearch/form)^25^, and AllergenFP v1.0 (https://ddg-pharmfac.net/AllergenFP/)^12^ were employed for allergy susceptibility analysis. The ToxinPred2 online service was employed in conjunction with default systems to estimate the toxicity of the epitope^26^. The TMHMM v2.0 (https://services.healthtech.dtu.dk/services/TMHMM-2.0/) method was employed to investigate the topology of the epitope^19^. Then, the immunogenicity of HTL epitopes was checked using the online servers IL4Pred (https://webs.iiitd.edu.in/raghava/il4pred/) ^29^, IL10Pred (https://webs.iiitd.edu.in/raghava/il10pred/)^30^ and IFNepitope (https://webs.iiitd.edu.in/raghava/ifnepitope/) ^31^.

### Epitope assembly, linkers, adjuvant integration and MEP vaccine design

The final MEP vaccine design was systematically revised to include probable T-cell and B-cell epitopes. The epitopes from cytotoxic T-cells (CTL), helper T-cells (HTL), and B-cells that met the established conditions constituted the final MEP vaccine design. To assemble the final MEP construct, specific linkers and adjuvants were strategically incorporated to optimize immunogenicity and structural flexibility. AAY linkers were inserted between CTL epitopes to promote efficient proteasomal cleavage and enhance MHC class I presentation. GPGPG linkers were used between HTL epitopes to ensure independent recognition and promote cytokine release, while KK linkers were placed between B-cell epitopes to maintain conformational stability and flexibility. Human β-defensin-3 was employed as an adjuvant at the N-terminal to stimulate Toll-like receptors (TLRs) and enhance innate immune activation. Additionally, a universal PADRE (Pan-DR epitope) sequence was included to overcome HLA polymorphism and strengthen CD4+ T-helper responses, thereby ensuring broad population coverage and improved vaccine efficacy. Finally, the EAAAK linker facilitated the connection between the adjuvant and the PADRE sequence.

### Physicochemical properties, antigenicity, allergenicity, topology, homology, and solubility analysis of the developed vaccine template

The vaccine’s physicochemical features were evaluated by inputting its FASTA sequence into the ProtParam online server of the ExPASy tools (https://web.expasy.org/protparam/) ^20^. The following characteristics were measured: the number of peptides, their aliphatic index, molecular weight, atomic composition, half-life estimation, extinction coefficients, instability index, calculated isoelectric point (pI), and the GRAVY (Grand Average of Hydropathicity). Subsequently, the VaxiJen v2.0 (https://www.ddg-pharmfac.net/vaxijen/VaxiJen/VaxiJen.html) was employed to predict the antigenicity of vaccines^18^. The AllergenFP v1.0 (https://ddg-pharmfac.net/AllergenFP/)^12^ and AllerTOP v2.0 (https://www.ddg-pharmfac.net/AllerTOP/)^23^ were employed to predict allergenicity due to the fact that allergenic proteins induce substantial immune responses. The topology of the vaccinations was analyzed using the TMHMM v2.0 (https://services.healthtech.dtu.dk/services/TMHMM-2.0/) approach^19^. The vaccine sequence was employed to analyze the homology properties using the NCBI BLASTp server (https://blast.ncbi.nlm.nih.gov/Blast.cgi?PROGRAM=blastp&PAGE_TYPE=BlastSearch&LINK_LOC=blasthome) along with the organisms, *Bos taurus* (taxid:9913) and *Bos indicus* (taxid:9915)^32^. Finally, the Protein-Sol server (https://protein-sol.manchester.ac.uk/) was employed to assess the solubility^33^.

### Secondary structure prediction

To generate the secondary (2D) structure examined by the SOPMA web server (https://npsa-prabi.ibcp.fr/cgi-bin/npsa_automat.pl?page=/NPSA/npsa_s opma.html) and the PSIPRED program (http://bioinf.cs.ucl.ac.uk/psipred/) ^34,35^. The web server uses two feed-forward neural algorithms to accurately analyze PSI-BLAST (https://blast.ncbi.nlm.nih.gov/Blast.cgi?PAGE=Proteins) protein content using default server settings throughout the trial.

### Tertiary structure prediction, refinement, and validation

The vaccine construct’s ultimate tertiary (3D) structure was generated with I-TASSER (https://zhanggroup.org/I-TASSER/)^36^. The model that was previously presumed was modified using the GalaxyRefine portal (http://galaxy.seoklab.org/cgibin/submit.cgi?type=REFINE)^37^. The updated models were then assessed by examining every model’s ERRAT value and Ramachandran plot using the SAVES v6.0 platform (https://saves.mbi.ucla.edu/) ^38,39^. The design’s overall performance regarding the Z-value was assessed using the ProSA web pages accessible at (https://prosa.services.came.sbg.ac.at/prosa.php)^40^.

### B-cell conformation epitope analysis

The ElliPro web server (http://tools.iedb.org/ellipro/) was used to anticipate that conformational B-cell epitopes would be cleaved inside the vaccination constructs^41^. The server parameters were used to generate epitope predictions using the default settings. These predictions provide essential insights into the possible immunogenic characteristics of the vaccine designs, therefore aiding the creation of more effective candidates for future experimental validation.

### Molecular Docking with Toll-like receptors (TLRs)

The RCSB protein data bank (https://www.rcsb.org/) was employed to acquire the PDB files for the receptors, with MEP vaccination functioning as the ligand^42^. The MEP vaccine construct’s affinity for binding to the surface toll-like receptors of *Bos taurus* (btTLR9, PDB ID: 3WPE) was determined through molecular docking using the ClusPro v2.0 webserver (https://cluspro.bu.edu/login.php)^43^. The energy calculation that yielded the lowest score was employed to select and retrieve the compounds that docked most professionally. Additionally, the ClusPro v2.0 webserver’s yield data was replicated using the most recent generation of the PyMOL v3.1 molecular visualisation utility^44^.

### Molecular dynamics (MD) simulation

Protein-protein complex microscopic stability is assessed using molecular dynamics, an advanced automated simulation method. Structure, function, fluctuation, interaction, and behavior are demonstrated to do this. The behavior of the one complex was investigated using GROMACS version 2025.1 for MD calculations^45^. CHARMM General Force Field parameterized protein content. The SwissParam (https://www.swissparam.ch/) server implemented ligand topologies. The structures were vacuum minimized 2500 times using steepest descent to address steric issues. The SPC water model solvated the structure. After adding Na + and Cl- ions, the gmx genion instrument balanced the system. This was done to ensure system electrical neutrality. After minimization, MD simulations went into production, NVT, and NPT. Two phases balanced the systems. First, a 100 picosecond NVT equilibration was done to maintain particle number, volume, and temperature. The procedure aimed to raise the system temperature to 300 K. The second stage involved a precise 100 picosecond NPT equilibration to achieve temperature, pressure, and particle number homogeneity. It was essential to maintain system density and pressure. The protein group’s location was limited by bond limitations on all bonds during simulations. The system entropy reduced because NVT and NPT restricted water molecules around the protein, relaxing them. Parrinello-Rahman barostat method and v-rescale thermostat for molecular dynamics^46^. The thermostat and barostat were adjusted for 100 picoseconds. To constrain covalent bonding, the Linear Constraint Solver application was used. Chemical bond interactions were handled using the sophisticated (Particle-Mesh Ewald) or PME approach. Every system has a 50-ns production run after equilibrium.

### Study on immune simulation

The C-ImmSim web server (http://150.146.2.1/C-IMMSIM/index.php) was employed to conduct the vaccine’s immunological simulation, which delivers real-time predictions of immune interactions^47,48^. With the exclusion of the time steps (1, 84, and 170) and the total number of replicated steps (1050), all parameters remained at their default values. The recommended immunization regimen includes three injections that are separated by four weeks, which is consistent with the dose intervals that are necessary for all marketable immunizations.

### Epitope assembly, linkers, and adjuvant integration

To assemble the final multi-epitope construct, specific linkers and adjuvants were strategically incorporated to optimize immunogenicity and structural flexibility. AAY linkers were inserted between CTL epitopes to promote efficient proteasomal cleavage and enhance MHC class I presentation. GPGPG linkers were used between HTL epitopes to ensure independent recognition and promote cytokine release, while KK linkers were placed between B-cell epitopes to maintain conformational stability and flexibility. Human β-defensin-3 was employed as an adjuvant at the N-terminal to stimulate Toll-like receptors (TLRs) and enhance innate immune activation. Additionally, a universal PADRE (Pan-DR epitope) sequence was included to overcome HLA polymorphism and strengthen CD4+ T-helper responses, thereby ensuring broad population coverage and improved vaccine efficacy^49^.

### Codon adaptation and *in Silico* cloning study

The AA sequence of the synthesized vaccines was sent to EMBOSS Backtranseq (https://www.ebi.ac.uk/Tools/st/embossbacktranseq/) to get cDNA for the optimization of codon use in *Escherichia coli*. The host can enhance the expression of foreign genes by codon optimization^50^. We utilized GenScript’s GenSmart codon optimization service (https://www.genscript.com/tools/gensmart-codon-optimization) to improve protein synthesis. The unconventional codon analysis tool from GenScript assessed the optimized construct. The host organism for this study was *E. coli*, and the vaccine cDNA sequence was provided to this service^51^. After this optimization, the ultimate sequence of the vaccine design was inverted, and the SnapGene v8.2.1 software was used to include HindIII and BamHI restriction sites at the N- and C-termini^52^.

## Results

### Protein sequence retrieval and comprehensive analysis

The protein sequences of the two proteins, dense granule protein 2 and surface protein Nc-p43, of NC that were chosen for this study were obtained from the NCBI database in the FASTA format. The appropriate antigenicity, transmembrane topology, and physicochemical properties of the two proteins are represented in Supplementary Table S1.

### B-cell epitope mapping with required analysis

The B-cell epitopes of the selected two proteins were predicted using the IEDB tools, based on six methods shown in Supplementary Figure S1 and Supplementary Table S2-S6. According to the analyses of antigenicity, allergenicity, toxicity, and topology, the two potential epitopes with minimal redundancy on B cells for both proteins are enumerated in Table 1.

**Table 1:** The potential selected B-cell epitopes for vaccine construction.

Epitope sequence	Antigenicity (> 0.4)	Allergenicity (non-allergen)	Toxicity (non-toxic)	Topology (outside)
NNRTLARRRRA	0.6013	Non-allergen	Non-toxic	Outside
WVALVYDSQ	2.2408	Non-allergen	Non-toxic	Outside

### T cell epitope mapping with comprehensive analysis

The epitopes of cytotoxic T-lymphocytes (CTL) and helper T-lymphocytes (HTL) for both proteins were predicted using NetMHC v4.0 and NetMHCII v2.3, respectively, based on the analysis of antigenicity, allergenicity, toxicity, topology, and immunogenicity, which includes IL4, IL10, and IFN-γ, as presented in Supplementary Table S3. Tables 2 and 3, respectively, provide the four potential epitopes with minimal redundancy for CTL and four potential epitopes with minimal redundancy for HTL from both proteins.

**Table 2:** The potential selected CTL epitopes for vaccine construction.

Epitope sequence	Antigenicity (> 0.4)	Allergenicity (non-allergen)	Toxicity (non-toxic)	Topology (outside)
GGENENGGE	2.0996	Non-allergen	Non-toxic	Outside
EVETDVQPS	1.6864	Non-allergen	Non-toxic	Outside
GCTGHPDDK	3.0160	Non-allergen	Non-toxic	Outside
GETGGENGD	2.8645	Non-allergen	Non-toxic	Outside

**Table 3:** The potential selected HTL epitopes for vaccine construction.

Epitope sequence	Antigenicity (> 0.4)	Allergenicity (non-allergen)	Toxicity (non-toxic)	Topology (outside)	IL4	IL10	IFN-γ
EEEAAEVETDVQPSS	1.4460	Non-allergen	Non-toxic	Outside	Inducer	Non-inducer	Non-inducer
VETDVQPSSVTIDTE	1.4202	Non-allergen	Non-toxic	Outside	Inducer	Non-inducer	Non-inducer
GLIVCNESDGEDECE	1.9834	Non-allergen	Non-toxic	Outside	Inducer	Non-inducer	Inducer
DDGLIVCNESDGEDE	1.6908	Non-allergen	Non-toxic	Outside	Inducer	Non-inducer	Inducer

### Multi-epitope peptide vaccine template design

**Fig. 2:**
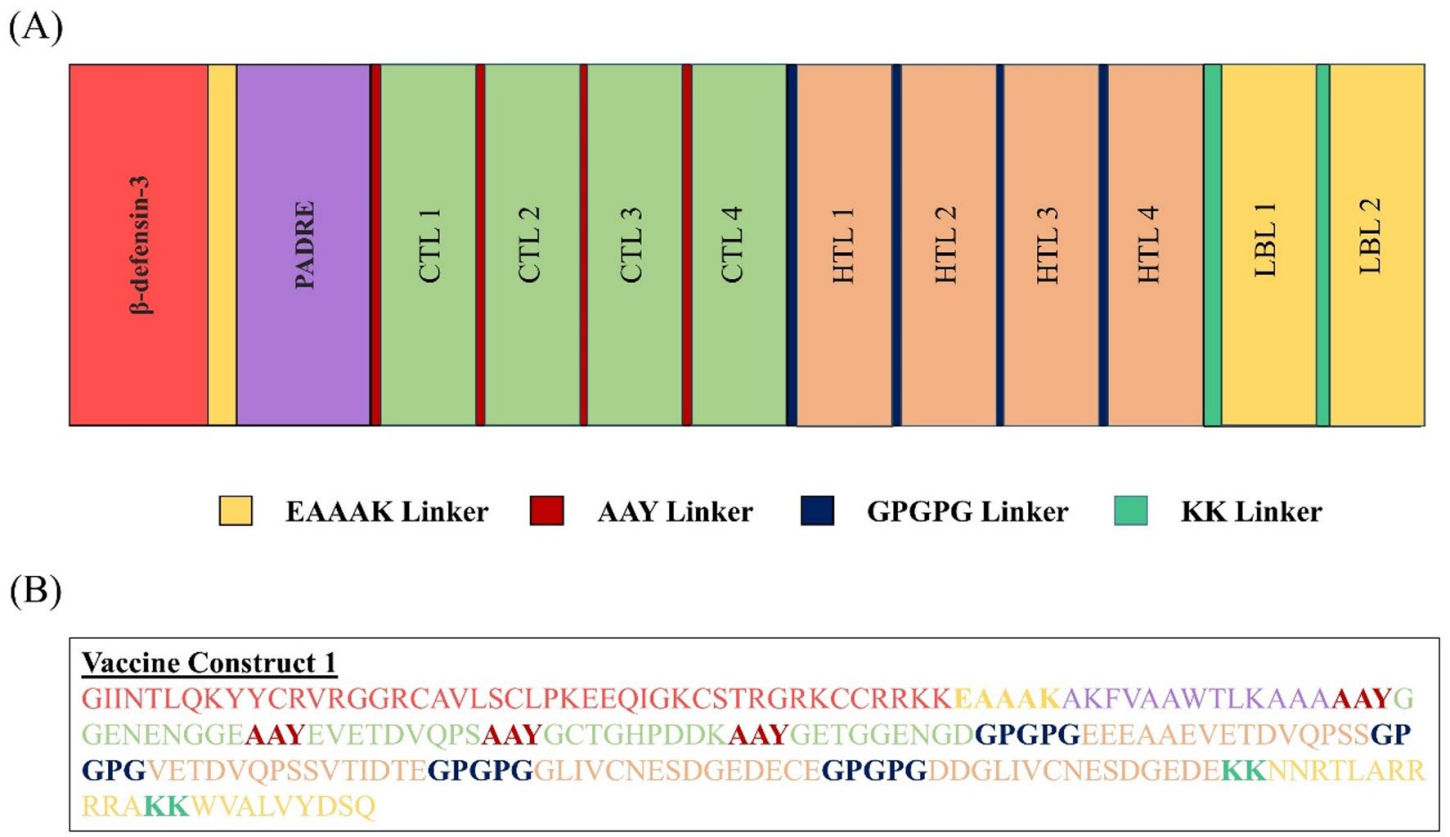
Designing of a MEP vaccine: (**A**) Schematic; (**B**) Constructive.

The approach focused on epitopes with significant antigenic potential that were non-toxic and non-allergenic. The vaccine’s design incorporates epitopes that fulfill these stringent criteria. β-defensin-3 was used to enhance the construct by using linkers such as AAY, GPGPG, and KK, ensuring precise positioning at the N-terminus to target T- and B-cell epitopes. The PADRE gene was used to enhance the vaccine’s overall immunogenicity. The construct included four CTLs, four HTLs, and two LBL epitopes. The construct has a total of 215 residues. Figure 2 presents a sequential and graphical depiction of the vaccine structures.

### Developed vaccine template analysis: physicochemical characteristics, antigenicity, allergenicity, topology, homology, and solubility

The physicochemical properties of the constructed vaccine were analysed using the ProtParam tool, revealing advantageous characteristics. The vaccine was anticipated to possess a pI of 4.71, indicating that it is very acidic. An estimated instability score of 39.67(< 40, stable) indicates that the vaccination comprises a stable protein. The vaccine exhibited hydrophobic characteristics, shown by a 54.98 aliphatic index and a GRAVY score of -0.782. The vaccine’s immunological efficacy was evaluated by examining the antigenicity score of 1.2156 by using the VaxiJen server. However, the external vaccine topology findings were predicted using TMHMM v2.0. Subsequently, BLASTp analysis revealed that the designed vaccine construct shared less than 25% query coverage with host proteins, indicating minimal sequence homology and reducing the risk of cross-reactivity. The vaccine’s solubility score of 0.948 indicated that its component was highly soluble. All findings are shown in Table 4.

**Table 4:** Antigenicity, allergenicity, toxicity, topology, solubility, sequence homology prediction and physico-chemical characteristics of the designed vaccine.

Features	Results
Antigenicity	1.2156
Allergenicity	Non-allergen
Transmembrane topology	Outside
Solubility	0.948 (soluble)
Sequence Homology	< 25% query coverage
Number of amino acids	215
Theoretical isoelectric point (pI)	4.71
Molecular Weight	22529.66
Formula	C_948_H_1503_N_283_O_334_S_10_
Total Number of atoms	3078
(Asp + Glu)	38
(Arg + Lys)	26
Half-life	30 h (mammalian reticulocytes, in vitro) > 20 h (yeast, in vivo) > 10 h (*Escherichia coli*, in vivo)
Aliphatic Index	54.98
Instability Index	39.67
GRAVY	− 0.782
Solubility	0.948

### Secondary structure prediction

**Fig. 3:**
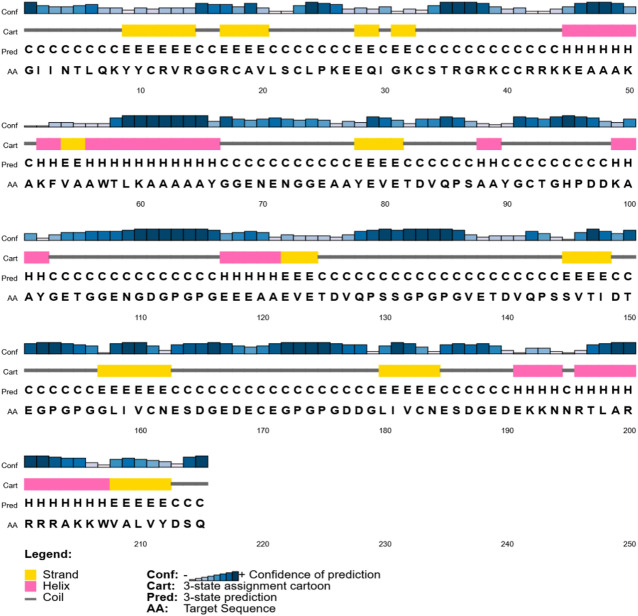
The PSIPRED server’s prediction of the vaccine’s secondary structure.

Two servers, PSIPRED and SOPMA, computed secondary (2D) structures, including extended strands, random coils, helices, and extended strands. Alpha helices (15.35%) and random coils (75.81%) are the most structurally complex, according to SOPMA. However, the extended strand comprises 8.84% of the structure. Complete secondary structure information is supplied by SOPMA in Table 5, and Fig. 3 illustrates the secondary structure of the vaccine, as specified by PSIPRED.

**Table 5:** Secondary structure of MEP vaccine according to SOPMA.

Secondary structure	Residues percentage
Random coils	75.81%
Alpha helices	15.35%
Extended strand	8.84%

### Prediction, refinement, and validation of tertiary structures

**Fig. 4:**
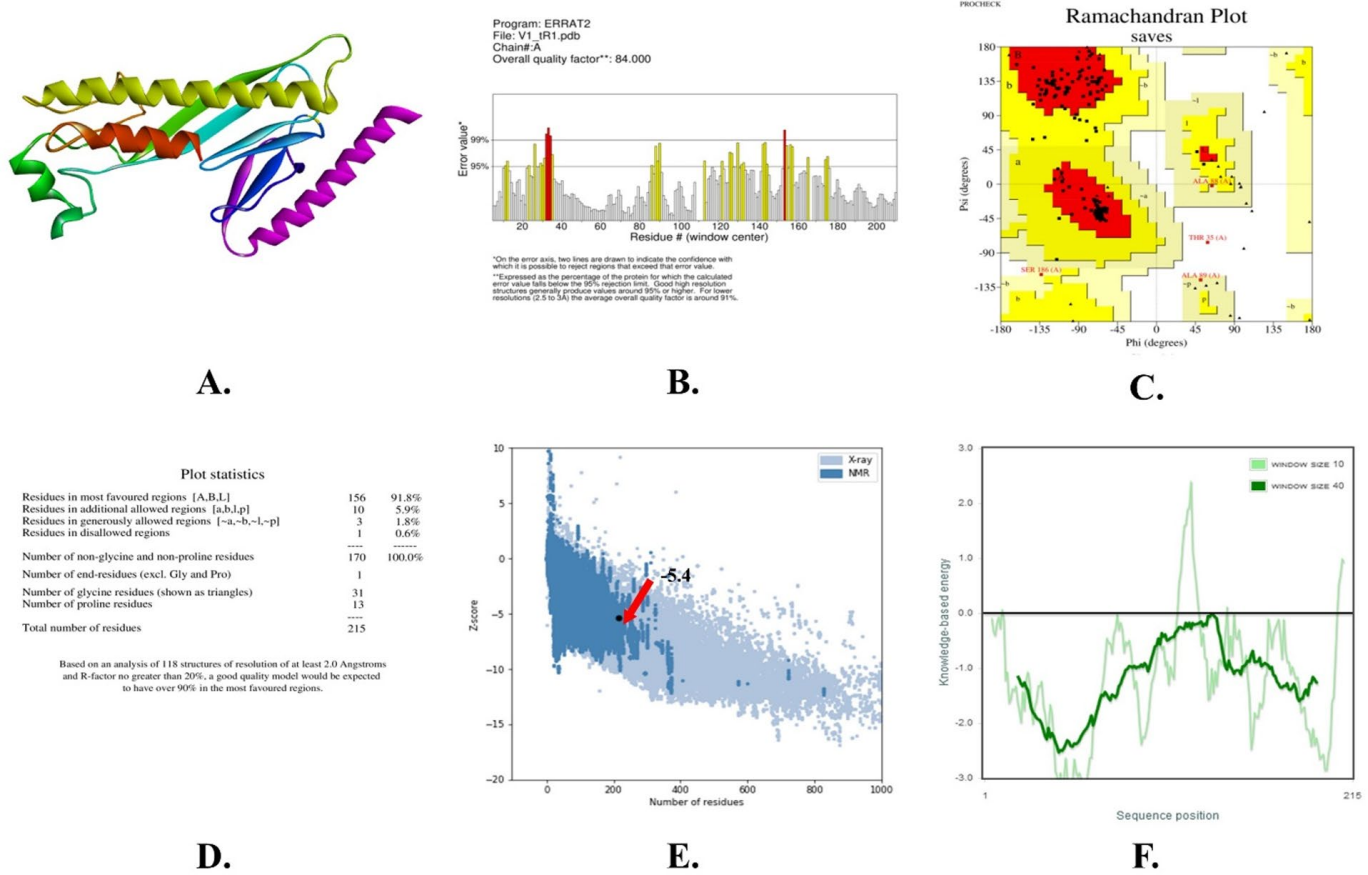
Forecasting the tertiary (3D) structure and confirming the vaccine design: (**A**) Three-dimensional model; (**B**) ERRAT quality score; (**C**) Ramachandran plot; (**D**) Ramachandran plot statistics; (**E**) Z-score graph; and (**F**) Z-score graph (sequence position).

We employed I-TASSER to generate the tertiary (3D) structure of the engineered vaccines. Subsequently, the vaccination model was optimized to achieve its highest level of precision by employing the online GalaxyWeb server. The optimal model was selected using the SAVESv6.0 server. Additionally, the ERRAT score of 84% was attained by the validated vaccination model. The Ramachandran plot analysis revealed that the model had a preferred region of 91.8% and a disallowed area of 0.6%. The Z-score was ascertained to be -5.4. ERRAT maps, Ramachandran plots, Z-score maps, and three-dimensional models of the vaccine construct are illustrated in Fig. 4.

### Prediction of B-cell conformational epitopes

**Fig. 5:**
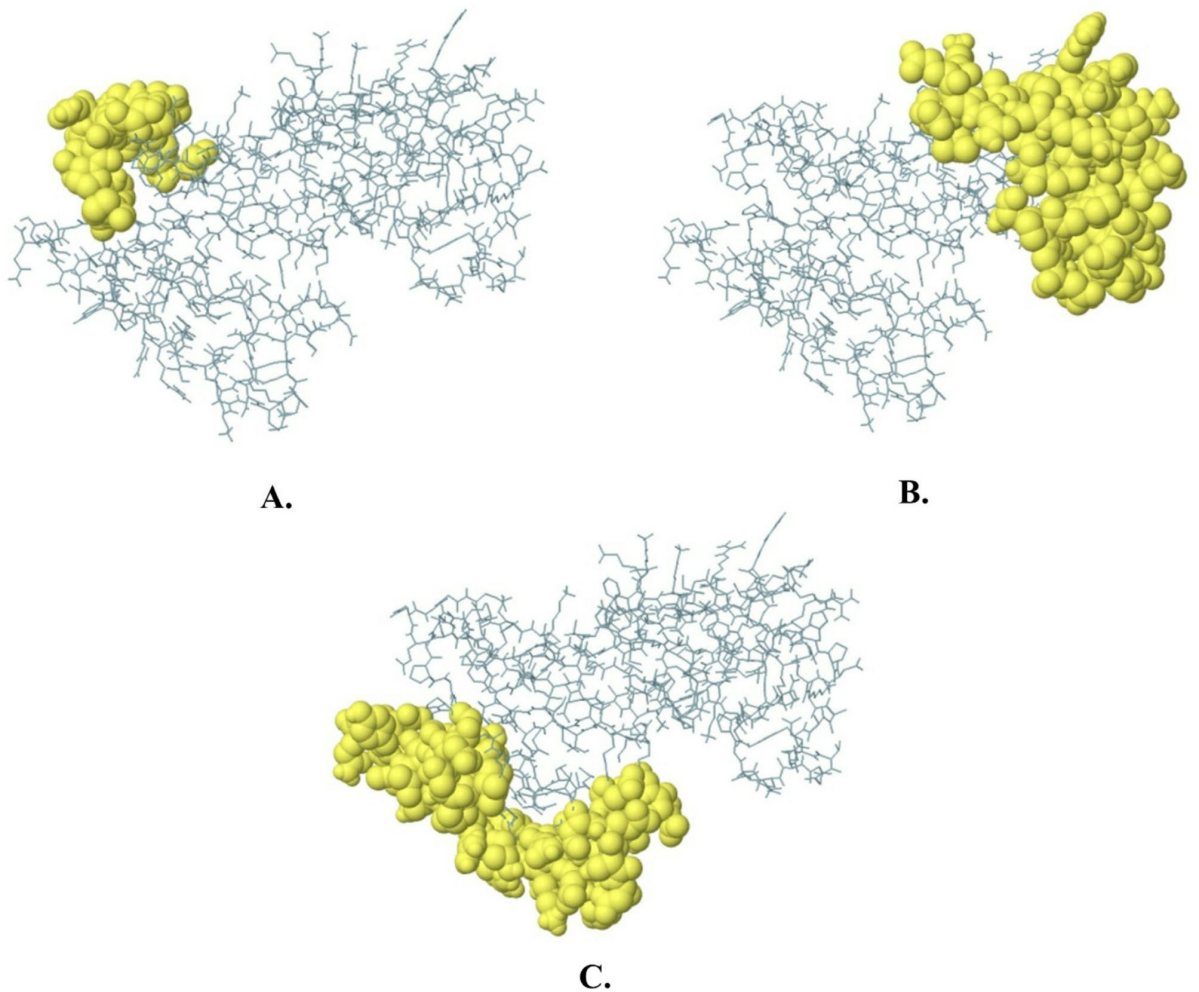
Predicted discontinuous B-cell epitopes mapped on the protein surface. The light-yellow spheres showing epitopes containing (**A**) 19 residues with 0.736; (**B**) 51 residues with 0.687; and (**C**) 47 residues with 0.673.

The ElliPro server from the IEDB database was utilized to predict discontinuous epitopes for NC vaccines. The Ellipro technique evaluates proteins’ Protrusion Index (PI) by analyzing their three-dimensional ellipsoidal shapes. The ElliPro server identified three discontinuous epitopes, with scores ranging from 0.673 to 0.736, as presented in Table 6. Therefore, these conformational epitopes are essential for inducing strong humoral immune responses, complementing the linear epitopes in the vaccine formulation. The 3D structure indicates the location of these epitopes on the protein surface and the accessibility for B-cell receptors to engage with them. Figure 5 shows discontinuous B-cell epitopes mapped on the protein surface.

**Table 6:** The potential selected B-cell conformation epitopes.

No.	Residues	Number of residues	Score
1	A: A64, A: G67–A: A77, A: S130–A: G134, A: G154–A: P155	19	0.736
2	A: R14–A: C18, A: L24–A: K39, A: C41, A: A88, A: Y90–A: E117	51	0.687
3	A: E151–A: P153, A: G156–A: L158, A: E163–A: D168, A: G174–A: D178, A: N184–A: T197, A: A199–A: R201, A: R203–A: Q215	47	0.673

### Molecular interaction with Toll-like receptors (TLRs)

**Fig. 6:**
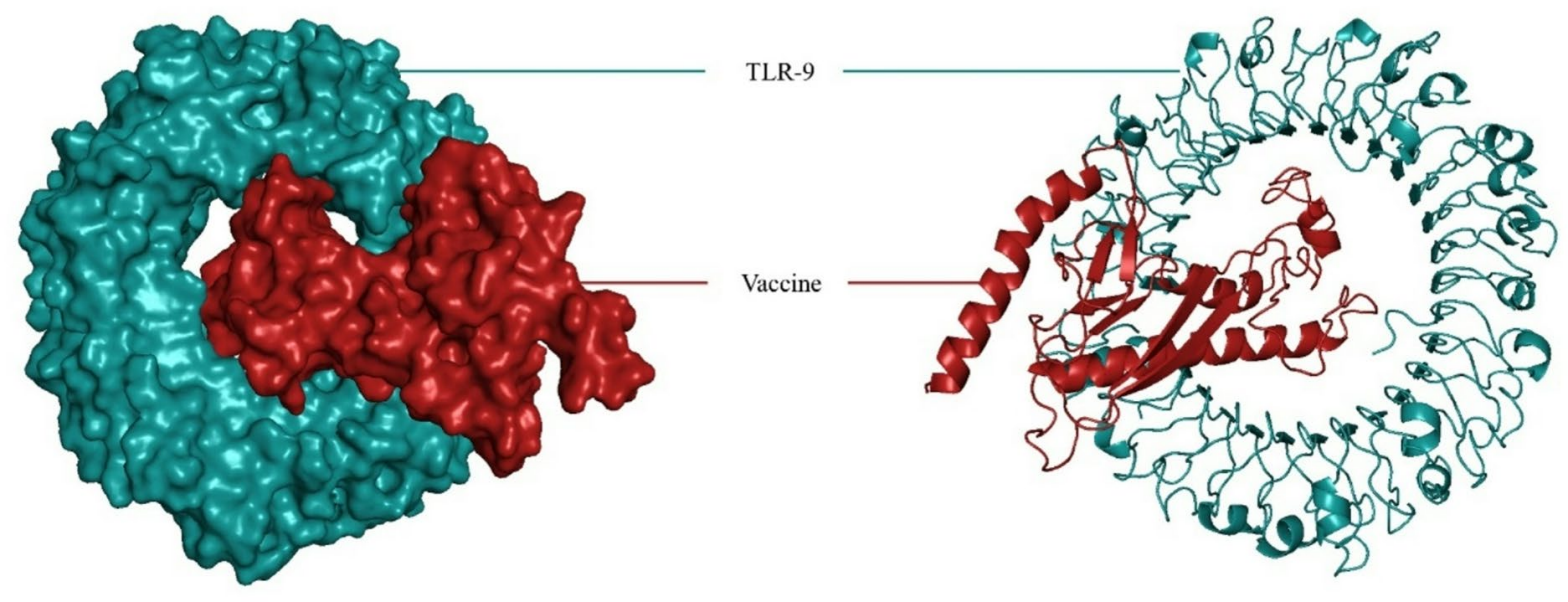
Three-dimensional (3D) models of TLR (Cyans)-Vaccine (Red) complex: *Bos taurus* TLR9 (PDB ID: 3WPE) with Vaccine.

The ClusPro v2.0 network was employed to bind molecules and evaluate the binding affinity of the MEP vaccine with the btTLR9 receptors. This is a crucial phase in the interaction between any vaccine candidate and the host immune receptor, resulting in a protective immunological response. The immunization strategy for the btTLR9 receptors produced energy scores of -1183.4. Figure 6 shows three-dimensional models of the btTLR9 vaccine complexes created by PyMOL.

### MD simulation

#### Root mean square deviation (RMSD)

**Fig. 7:**
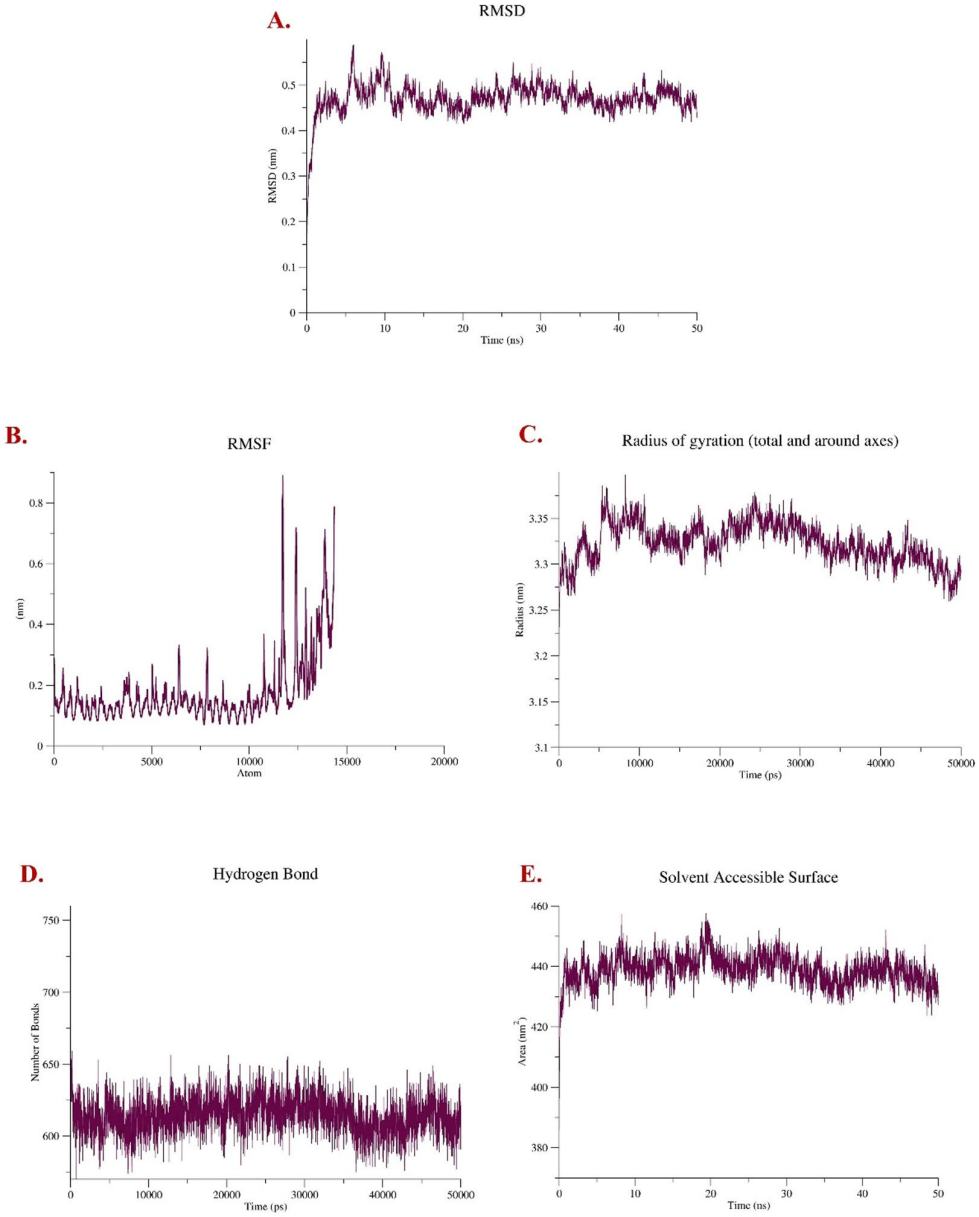
MD simulation of the btTLR9-vaccine complex: (**A**) Root-Mean-Square Deviation (RMSD); (**B**) Root-Mean-Square Fluctuation (RMSF); (**C**) Radius of Gyration (Rg); (**D**) Solvent Accessible Surface Area (SASA); and (**E**) Hydrogen bond formation.

The RMSD (Root Mean Square Deviation) plot of the protein backbone reflects the time-dependent deviation of the protein structure from its initial conformation during the 50 ns molecular dynamics simulation. A sharp rise in RMSD within the first 2–3 nanoseconds indicates that the receptor–vaccine complex rapidly underwent conformational reorganization likely due to relaxation from its initial, potentially strained or energetically suboptimal docked state. This behavior is common as the system minimizes energy and finds a thermodynamically favorable conformation, illustrated in Fig. 7a. Following this initial adaptation phase, the RMSD values plateau around 0.45 nm, signifying that the backbone of the protein maintains a relatively stable tertiary structure throughout the remainder of the simulation. The narrow fluctuation range of 0.40–0.50 nm suggests there is no large-scale unfolding or significant conformational disruption, which is indicative of a robust and well-maintained binding interaction with the vaccine.

#### Root mean square fluctuation (RMSF)

The RMSF (Root Mean Square Fluctuation) analysis provides insight into the per-residue flexibility of the protein throughout the simulation. Most residues display RMSF values below 0.25 nm, indicating that the majority of the protein maintains a relatively rigid and stable conformation depicted in Fig. 7b. This structural restraint reflects strong intramolecular interactions such as backbone hydrogen bonding and secondary structure elements (α-helices and β-sheets) that confer conformational stability upon vaccine binding. Notably, higher fluctuations are observed in select regions, particularly near the C-terminal domain, where RMSF peaks exceed 0.6 to 0.9 nm. These elevated values likely correspond to flexible loop regions or unstructured terminal ends, which are inherently more dynamic due to the lack of stabilizing secondary structure. From a chemical and structural biology perspective, such localized flexibility is expected and may play a role in biological function, such as vaccine access, conformational switching, or protein–protein interactions. The absence of dramatic fluctuations across the protein’s core reaffirms that vaccine binding does not destabilize the complex, supporting the integrity of the protein’s global fold during the simulation timeframe.

#### Radius of gyration (Rg)

The Radius of Gyration (Rg) plot provides a measure of the overall compactness and mass distribution of the protein–protein complex over time. In this case, the Rg profile remains relatively steady around an average value of ~ 3.3 nm throughout the 50 ns molecular dynamics simulation mentioned in Fig. 7c. This minimal fluctuation implies that the global structure of the protein remains tightly packed and does not undergo significant expansion, partial unfolding, or structural collapse. From a molecular perspective, the slight initial rise in Rg likely corresponds to conformational relaxation as the system transitions from an energy-minimized state to an equilibrated ensemble under physiological conditions. Once stabilized, the consistent Rg trend signifies that the protein’s tertiary structure is preserved, with no major disruptions to its hydrophobic core or secondary structural elements. Together with the RMSD and SASA results, the stable Rg profile chemically validates that the presence of the vaccine does not induce destabilizing effects, and the complex retains a well-folded, thermodynamically favorable conformation throughout the simulation duration.

### Hydrogen bonds

Intramolecular hydrogen bonding was evaluated using the gmx hbond utility in GROMACS 2025.1 to assess the structural stability of the protein-protein complex during a 100 ns molecular dynamics simulation. The analysis applied standard geometric criteria hydrogen bond distance ≤ 0.35 nm and donor–hydrogen–acceptor angle ≥ 120° to identify and quantify hydrogen bonds in each frame of the trajectory shown in Fig. 7e. This approach enables the detection of subtle structural perturbations and helps infer the integrity of the protein’s internal network.

Throughout the simulation, the number of hydrogen bonds fluctuated between 580 and 670, maintaining a consistent average of approximately 610–620. From a chemical perspective, these high and stable counts indicate a well-preserved hydrogen bonding network that stabilizes the protein’s tertiary structure. The lack of a significant increase or decrease in hydrogen bond count across time also implies that the binding of the vaccine does not compromise the internal folding or integrity of the protein. Instead, it reinforces a thermodynamically stable and conformationally resilient complex, supporting sustained non-covalent interactions and a biologically plausible binding mode.

### Solvent accessible surface area (SASA)

The Solvent Accessible Surface Area (SASA) plot reveals how much of the protein surface is exposed to solvent throughout the simulation. In this analysis, the SASA values range from approximately 420 to 450 nm², showing only minor fluctuations. These small variations are indicative of natural breathing motions or slight conformational adjustments in flexible surface regions, rather than any major structural rearrangements or unfolding events illustrated in Fig. 7d.

From a chemical standpoint, the relatively high and consistent SASA values suggest that the complex remains moderately exposed to solvent, which aligns with a ligand such as vaccine binding at a site near or partially on the protein surface. This surface accessibility is functionally relevant, as peptide vaccines often interact with surface-exposed grooves or shallow binding pockets rather than deep, buried active sites. Overall, the stable SASA profile supports the structural integrity of the complex and corroborates the inference that vaccine binding does not trigger significant conformational destabilization or solvent shielding throughout the trajectory.

### Study on immune simulation

**Fig. 8:**
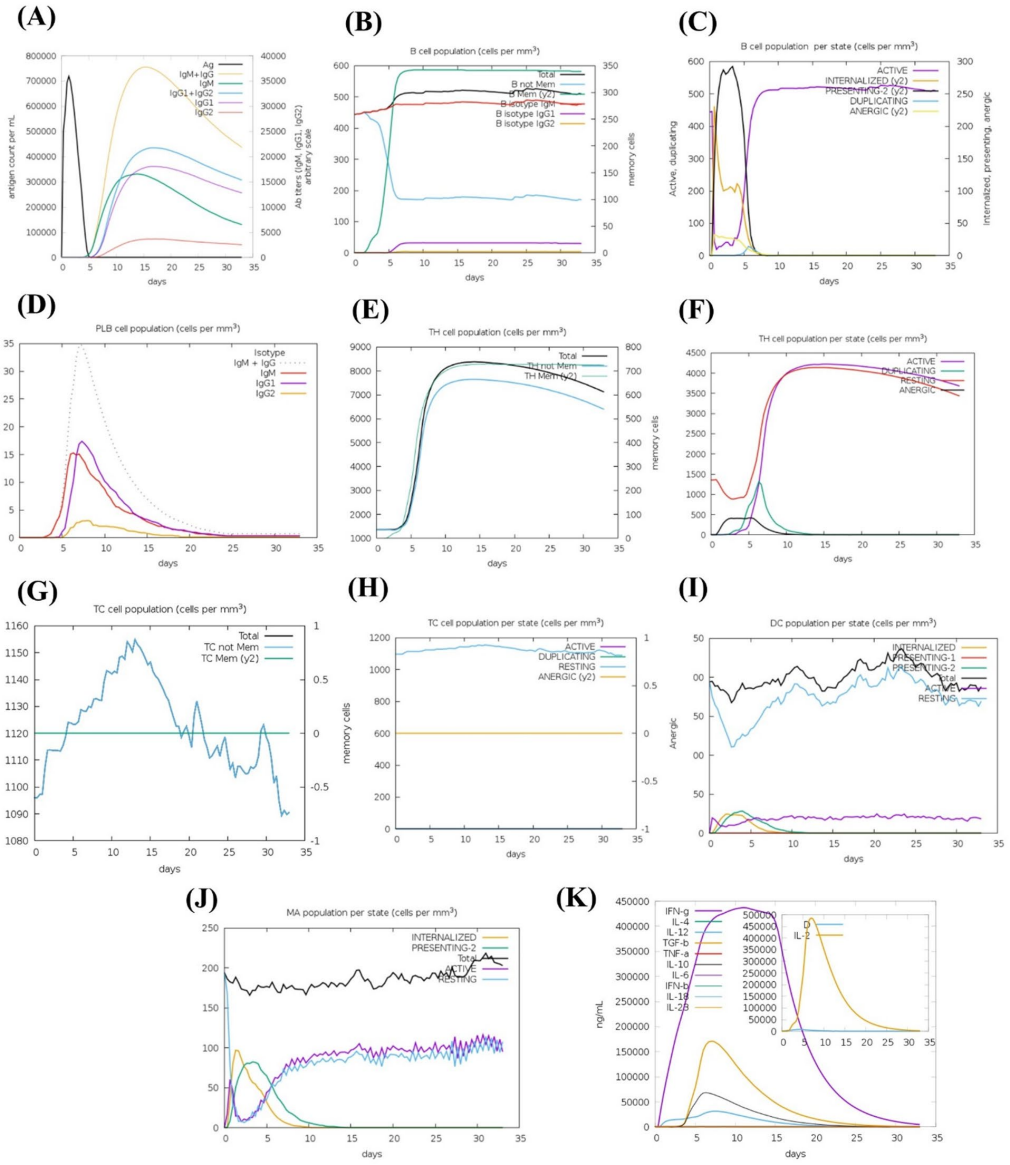
C-ImmSimm illustrates the immunological activation and diverse immune responses generated by the engineered immunizations during three doses. (**A**) The black lines depict the effect of vaccine immunizations on immunoglobulins and immune complexes, whilst the colored lines represent the different subclasses of these immunoglobulins; (**B**) This illustrates the proliferation of the B cell population; (**C**) The B-cell population fluctuates in accordance with the state during the immunization procedure; (**D**) The number of plasma B-cells (PLBs) increases during the injection procedure; (**E**) The overall number of helper T-cells (TH) increased after three administrations; (**F**) The total number of helper T-cells produced in all conditions subsequent to immunization; (**G**) The activity of regulatory T cells (TC) increased after three administrations; (**H**) The infusions resulted in an increase in the cytotoxic T-cell count; (**I**) The total number of active cytotoxic T-cells in each stage increased after three treatments; DC denotes dendritic cells; (**J**) The total number of macrophages (MP) and active dendritic cells (DC) expanded in all conditions throughout the three administrations; and (**K**) The levels of several cytokines increased over the course of the three dosages.

Immunological simulations demonstrated that vaccination may generate immune responses that support the body’s defenses. Figure 8A shows that following injection, antigen (Ag) concentration increases, IgM is generated initially, and after booster doses, IgG1 and IgG2 dominate, suggesting class switching and a greater memory response. In contrast, Fig. 8B shows that B cell subtypes (naïve, active, memory, and isotype-switched) exhibit regulated immune responses and contribute to long-term immunological memory. Figure 8C demonstrates the durability of memory B-cell immunity. In Fig. 8D, plasma cells secrete most antibodies. However, effector and memory TH cells (Fig. 8E) seem to be essential for B cell activation, antibody switching, and cytotoxic T cell stimulation. Figure 8F also reveals a rise in active TH cells, indicating robust antigen recognition, immunological coordination, and persistent memory. Thereafter, cytotoxic T cells-maintained memory and increased activity, indicating a cell-mediated immune response (Fig. 8G and H). DCs are antigen-presenting cells (APCs) that efficiently prime T cells and increase presenting DCs, confirming vaccine uptake and immune system activation (Fig. 8I). After a large initial increase in antigen processing and presentation, macrophage populations remained active and resting, indicating persistent innate immune activation (Fig. 8J). Cytokine analysis showed that IFN-γ, IL-2, and IL-12 levels increased post-vaccination and decreased over time, but IL-10 remained stable (Fig. 8K). Figure 8 depicts the outcome.

Molecular Mechanics Poisson-Boltzmann Surface Area (MM-PBSA) calculations were performed using the g-mmpbsa package in GROMACS to estimate binding free energies between the vaccine construct and TLR9 receptor.

### In Silico cloning and codon adaptation study

**Fig. 9:**
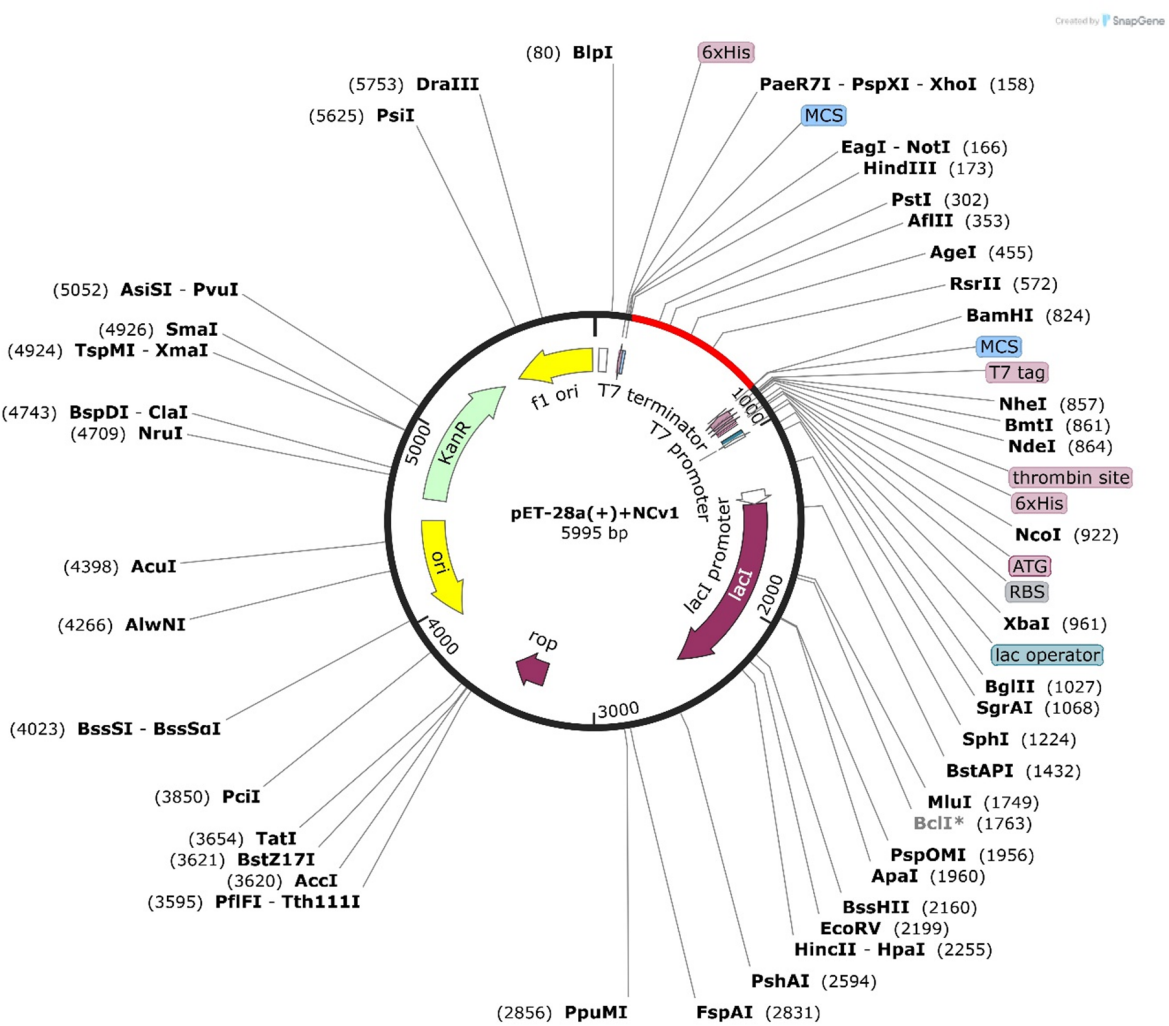
Codon adaptation and in-silico cloning of the vaccine construct.

The Codon Adaptation Index (CAI) indicates that the vaccine’s modified codons account for a greater proportion of the most frequently occurring codons. The elevated GC content of 58.76 and CAI value of 0.99519 were used to identify the altered codons. The absence of restriction sites in HindIII and BamHI was verified to ensure the safety of the cloning operation. The pET28a (+) vector, which contained the HindIII and BamHI restriction regions, was subsequently inserted with the optimized codons. As a result, the pET28a (+) vector was implemented to produce a 5995 bp clone. In Fig. 9, the desired region is illustrated in red between each pET28a (+) vector sequence.

## Discussion

NC is the causative agent of neosporosis, a multisystemic disease described as an obligate intracellular, tissue cyst-forming coccidian parasite, which significantly contributes to reproductive losses in cattle and neuromuscular diseases in dogs worldwide^53^. *NC* has no permanent vaccination due to physical and biological characteristics similar to those of its close relative^54^. Consequently, this vaccination is the most effective approach for avoiding the transmission of zoonotic NC and controlling neosporosis in cattle and other animals^55^. In this study, we developed an innovative MEP vaccine construct targeting NC through the application of immunoinformatics methodologies. The vaccine formulation incorporated epitopes derived from dense granule protein 2 (GRA2) and surface antigen Nc-p43, both of which are recognized for their significant contributions to the antigenicity and virulence of NC. In the past, the research conducted by Pinitkiatisakul revealed that a recombinant vaccination offered partial protection against NC in mice^14^. Recent advancements in bioinformatics techniques have enabled the development of novel vaccine design methodologies, allowing researchers to accurately forecast epitope peptides that could serve as vaccines to elicit immunological responses^56^. Our findings are consistent with earlier studies that applied reverse vaccinology for epitope-based vaccines against *Toxoplasma gondii*^57^ and *Streptococcus agalactiae*^28^, but extend this work by targeting both GRA2 and Nc-p43 proteins simultaneously, thereby enhancing the potential breadth of immune protection. Recombinant NcSRS2 vaccines provided partial protection in cattle, while live-attenuated Nc-1 strain showed improved efficacy but raised safety concerns^15^. Our in-silico design aims to address these limitations by offering a safer, epitope-focused approach. Immunoinformatics approaches have produced epitope-based vaccinations for *Toxoplasma gondii* [12], SARS-CoV-2 ^58^, Nipah henipavirus^59^, hepatitis B virus^60^, and fowlpox virus^61^. Conversely, the primary objective of our MEP vaccine is to elicit immune responses that provide improved protection and eradicate this pathogenic parasite.

The dense granule protein 2 (GRA2) and surface antigen Nc-p43 protein are key targets owing to their elevated antigenicity shown in physicochemical analyses, making them appropriate for MEP vaccine development. This work employs NC protein B-cell-restricted and T-cell epitopes to enhance immunity, using known parameters like antigenicity, allergenicity, toxicity, topology, immunogenicity, and the cytokines IFN-γ, IL-4, and IL-10 (applicable only to HTL epitopes). The amalgamation of epitopes with the adjuvant β-defensin-3, PADRE, and linkers (AAY, GPGPG, KK) improved the efficacy of vaccination. Similar adjuvant-linked MEP vaccine designs have been successfully reported in computational vaccine studies against SARS-CoV-2^62^ and Nipah virus^59^, reinforcing the robustness of this strategy.

Human β -defensin 3 augments antigen presentation and Th1 responses in animal models of Toxoplasma and Plasmodium, functioning as an adjuvant^12,62,63^. The computational research indicated that the vaccine exhibits strong antigenicity, non-toxicity, non-allergenic qualities, structural stability, TMHMM topology, protein solubility, and ExPasy’s ProtParam physicochemical features, indicating its suitability for experimental validation.

The vaccine construct exhibited significant antigenicity, achieving a score of 1.2156. This vaccine has more antigenicity than the alternative MEP vaccine designed for NC, which was reported to be 0.9502 ^13^. The vaccine elicited a robust immunogenic response, as evidenced by an increase in antigen-specific B cells, helper T cells, cytotoxic T cells, and memory cells, as demonstrated by immunological simulation studies. Additionally, the enhanced level of cytokines, including IL-4, IL-10, and IFN-γ, indicates a well-regulated immune response, essential for the prevention of NC infection. NC infection triggers a robust Th1-type immune response in cattle and dogs, mostly mediated by IFN-γ and IL-10 ^53,64^. An effective vaccination ought to strengthen this response while reducing suppression. The vaccine design utilizes IL-4 inducer epitopes to elicit a balanced immune response, IL-10 non-inducer epitopes to prevent immune suppression, and IFN-γ inducers to augment cellular immunity, therefore reinforcing protection against NC ^62^.

The vaccine’s 3D structure has been predicted, improved, and verified to improve its capacity to interact with TLRs, which are imperative for the initiation of the innate immune response^60^. Toll-like receptors (TLRs) are essential receptor proteins that interact with the nucleic acids and envelope proteins of infections to identify pathogens through Pathogen-Associated Molecular Patterns (PAMPs) and provoke an immune response^60^. This research project explored the interaction between the vaccine design and a single critical TLR (btTLR9). Comparable stability patterns were observed in epitope-based vaccines against *Toxoplasma gondii*^66^ and SARS-CoV-2 ^67^, suggesting that our construct demonstrates reliability and translational potential similar to previously validated designs. Unlike earlier single-protein based vaccine studies^14^, our dual-target approach potentially reduces the risk of immune escape and broadens host immune coverage.

Molecular dynamics simulations revealed that the receptor-vaccine complex remains structurally stable under physiological conditions. The initial rise in RMSD during the first 2–3 ns likely corresponds to a conformational relaxation from the docked state, as the system seeks a lower-energy configuration. The subsequent stabilization around 0.45 nm reflects a well-maintained tertiary structure, suggesting that the vaccine does not induce global unfolding or instability. RMSF analysis showed that most residues remain conformationally constrained (RMSF < 0.25 nm), particularly in the protein’s core. Expectedly, elevated fluctuations are localized at the C-terminal and loop regions areas typically associated with greater flexibility. This localized mobility is not indicative of instability but may support biological functions such as vaccine accommodation or molecular recognition. The Radius of Gyration (Rg) remained consistent around ~ 3.3 nm, indicating that the protein’s compactness and overall fold are preserved throughout the simulation. Together with RMSD and RMSF profiles, the stable Rg values suggest that vaccine binding does not disrupt the global structure or hydrophobic core. SASA values remained within 420–450 nm², showing only minor surface fluctuations. These changes are characteristic of natural breathing motions rather than structural collapse. The moderate and stable SASA profile implies that the vaccine likely binds to a partially solvent-exposed pocket, consistent with its nature as a peptide vaccine. Importantly, the hydrogen bond network was preserved, with an average of 610–620 intramolecular hydrogen bonds maintained throughout the 100 ns simulation. This stability further confirms the absence of significant structural perturbation upon vaccine binding. Overall, the results support that vaccine binding induces no destabilizing effects, preserving the native structure and internal interactions of the protein. These findings provide a solid foundation for further investigation in vaccine design.

The optimization of codons and the In-silico cloning into the *E. coli* expression system revealed advantageous parameters for recombinant expression, characterized by a high Codon Adaptation Index (CAI) and appropriate GC content. This study demonstrates novelty by integrating GRA2 and Nc-p43 antigens into a MEP vaccine construct, supported by docking and MD evidence comparable to successful immunoinformatics vaccines reported for other pathogens. This represents a pivotal step towards the future validation of experiments and the large-scale production of vaccines. Notwithstanding the encouraging outcomes derived from this study, it is imperative to recognize certain limitations. The In-silico predictions, although significantly informative, necessitate additional in vitro and in vivo validation to ascertain the vaccine’s efficacy and immunogenic potential. Future research endeavors should prioritize the expression of recombinant proteins, the assessment of immunogenicity through animal models, and the execution of challenge experiments to evaluate the protective efficacy against NC infection.

## Conclusions

This work focused on the pathogenic GRA2 and Nc-p43 proteins as targets for in silico multi-epitope peptide (MEP) vaccine against NC. Although NC mostly impacts animals, especially cattle, it raises concerns over potential zoonotic transmission. The vaccine targets conserved antigenic areas vital for the parasite’s existence, perhaps providing protective immunity if the parasite can infect cattle. Nonetheless, in silico predictions may not fully represent biological complexity; hence, experimental validation is necessary to confirm effectiveness, expression, and safety. Future studies should assess the cross-protective potential of protozoan protection in cattle, explore therapeutic applications, and enhance vaccine administration using genetic engineering and nanotechnology.

## Supplementary Information

Below is the link to the electronic supplementary material.

Supplementary Material 1

## Data Availability

The data supporting the findings of this study can be obtained from the corresponding authors upon reasonable request.

## References

[1] Fayisa, W. O. A current update on *Neospora Caninum*. *Microbiol. Res. J. Int.* **33**, 32–37 (2023).

[2] Rimayanti, R. et al. Review of neosporosis: disease insights and control approaches. *Print) Rev. Article ISSN*. **15**, 2218–6050 (2025). 10.5455/OVJ.2025.v15.i3.2 PMC12017711 40276172

[3] Dubey, J. P., Schares, G. & Ortega-Mora, L. M. Epidemiology and control of neosporosis and *Neospora Caninum*. *Clin. Microbiol. Rev.* **20**, 323–367 (2007). 17428888 10.1128/CMR.00031-06 PMC1865591

[4] Maia, M. O. et al. Seroprevalence of *Toxoplasma gondii* and *Neospora Caninum* in sheep intended for human consumption in the Rondônia state, Western Brazilian Amazon. *Comp Immunol. Microbiol. Infect. Dis* **74**, (2021). 10.1016/j.cimid.2020.101599 33260021

[5] Davidson, M. J. et al. Active shedding of *Neospora Caninum* detected in Australian wild Canids in a nonexperimental context. *Transbound. Emerg. Dis.* **69**, 1862–1871 (2022). 34043877 10.1111/tbed.14170 PMC9542884

[6] Barimani, S., Rassouli, M. & Emadi Chashmi, S. H. Molecular detection of *Neospora Caninum* in chicken meat and eggs in Iran. *Vet. Parasitol. Reg. Stud. Rep.* **40**, 100862 (2023). 10.1016/j.vprsr.2023.100862 37068865

[7] Shahiduzzaman, M. et al. First report of *Neospora Caninum* from aborted fetuses of cattle, sheep, and goats in Bangladesh. *J Adv. Vet. Anim. Res* **11**, (2024). 10.5455/javar.2024.k811 PMC11590592 39605763

[8] Mohammed, R. R., Tavassoli, M., Sidiq, K. R. & Esmaeilnejad, B. Prevalence of *Neospora Caninum* as an etiologic agent of animal abortion in Kurdistan region of Iraq. *Pol. J. Vet. Sci.* **26**, 349–357 (2023). 37727044 10.24425/pjvs.2023.145039

[9] Yang, C., Wang, C., Liu, J. & Liu, Q. Biotinylation of the *Neospora Caninum* parasitophorous vacuole reveals novel dense granule proteins. *Parasit. Vectors*. **14**, 1–12 (2021). 34625097 10.1186/s13071-021-05023-7 PMC8501707

[10] Dong, J. et al. Disruption of dense granular protein 2 (GRA2) decreases the virulence of *Neospora Caninum*. *Front. Vet. Sci.* **8**, 634612 (2021). 33681332 10.3389/fvets.2021.634612 PMC7933011

[11] Goodswen, S. J., Kennedy, P. J. & Ellis, J. T. Discovering a vaccine against neosporosis using computers: is it feasible? *Trends Parasitol.* **30**, 401–411 (2014). 25028089 10.1016/j.pt.2014.06.004

[12] Ahmed, N. et al. Designing a multi-epitope subunit vaccine against *Toxoplasma gondii* through reverse vaccinology approach. *Mol. Biochem. Parasitol.* **260**, 111655 (2024). 39521441 10.1016/j.molbiopara.2024.111655

[13] Shams, M. et al. Towards the First Multiepitope Vaccine Candidate against *Neospora caninum* in Mouse Model: Immunoinformatic Standpoint. *Biomed Res Int* (2022). (2022). 10.1155/2022/2644667 PMC9204498 35722460

[14] Pinitkiatisakul, S. Recombinant subunit vaccines against *Neospora Caninum*. *Acta Universitatis Agriculturae Sueciae* (2007). https://res.slu.se/id/publ/14262

[15] Reichel, M. P. et al. A live vaccine against *Neospora Caninum* abortions in cattle. *Vaccine* **33**, 1299–1301 (2015). 25659274 10.1016/j.vaccine.2015.01.064

[16] Cannas, A. et al. Vaccination of mice against experimental *Neospora Caninum* infection using NcSAG1- and NcSRS2-based Recombinant antigens and DNA vaccines. *Parasitology* **126**, 303–312 (2003). 12741509 10.1017/s0031182002002895

[17] Sayers, E. W. et al. Database resources of the National center for biotechnology information. *Nucleic Acids Res.* **49**, D10–D17 (2021). 33095870 10.1093/nar/gkaa892 PMC7778943

[18] Doytchinova, I. A. & Flower, D. R. VaxiJen: a server for prediction of protective antigens, tumour antigens and subunit vaccines. *BMC Bioinform.* **8**, 1–7 (2007). 10.1186/1471-2105-8-4 PMC1780059 17207271

[19] Krogh, A., Larsson, B., Von Heijne, G. & Sonnhammer, E. L. L. Predicting transmembrane protein topology with a hidden Markov model: application to complete genomes. *J. Mol. Biol.* **305**, 567–580 (2001). 11152613 10.1006/jmbi.2000.4315

[20] Gasteiger, E. et al. *Protein Identification and Analysis Tools on the ExPASy Server* (Springer, 2005). 10.1385/1-59259-584-7:531 10027275

[21] Vita, R. et al. The immune epitope database (IEDB): 2018 update. *Nucleic Acids Res.* **47**, D339–D343 (2019). 30357391 10.1093/nar/gky1006 PMC6324067

[22] Mohmed, R., Ahmed, S., Almofti, Y. A. & Ali, K. Analysis of foot and mouth disease virus polyprotein for multi peptides vaccine design: an In-silico strategy. **16**, 2083–2098 (2022).

[23] Dimitrov, I., Bangov, I., Flower, D. R. & Doytchinova, I. AllerTOP v.2 - A server for in Silico prediction of allergens. *J. Mol. Model.* **20**, 1–6 (2014). 10.1007/s00894-014-2278-5 24878803

[24] Goodman, R. E. et al. AllergenOnline: A peer-reviewed, curated allergen database to assess novel food proteins for potential cross-reactivity. *Mol. Nutr. Food Res.* **60**, 1183–1198 (2016). 26887584 10.1002/mnfr.201500769

[25] Fiers, M. W. E. J. et al. Allermatch™, a webtool for the prediction of potential allergenicity according to current FAO/WHO Codex alimentarius guidelines. *BMC Bioinform.* **5**, 1–6 (2004). 10.1186/1471-2105-5-133 PMC522748 15373946

[26] Sharma, N., Naorem, L. D., Jain, S. & Raghava, G. P. S. ToxinPred2: an improved method for predicting toxicity of proteins. *Brief. Bioinform*. **23**, bbac174 (2022). 35595541 10.1093/bib/bbac174

[27] Reynisson, B., Alvarez, B., Paul, S., Peters, B. & Nielsen, M. NetMHCpan-4.1 and NetMHCIIpan-4.0: improved predictions of MHC antigen presentation by concurrent motif Deconvolution and integration of MS MHC eluted ligand data. *Nucleic Acids Res.* **48**, W449–W454 (2020). 32406916 10.1093/nar/gkaa379 PMC7319546

[28] Pathak, R. K., Lim, B., Kim, D. Y. & Kim, J. M. Designing multi-epitope-based vaccine targeting surface Immunogenic protein of *Streptococcus agalactiae* using immunoinformatics to control mastitis in dairy cattle. *BMC Vet. Res.* **18**, 1–17 (2022). 36071517 10.1186/s12917-022-03432-z PMC9449294

[29] Dhanda, S. K., Gupta, S., Vir, P. & Raghava, G. P. S. Prediction of IL4 inducing peptides. *Clin Dev Immunol* (2013). (2013). 10.1155/2013/263952 PMC3893860 24489573

[30] Singh, O., Hsu, W. L. & Su, E. C. Y. Ileukin10pred: A computational approach for predicting il-10-inducing immunosuppressive peptides using combinations of amino acid global features. *Biology (Basel)*. **11**, 5 (2022). 10.3390/biology11010005 PMC8773200 35053004

[31] Huang, J., Cao, Y., Bu, X. & Wu, C. Residue analysis of a CTL epitope of SARS-CoV Spike protein by IFN-gamma production and bioinformatics prediction. *BMC Immunol.* **13**, 1–10 (2012). 22963340 10.1186/1471-2172-13-50 PMC3575293

[32] Stover, N. A. & Cavalcanti, A. R. O. Using NCBI BLAST. *Curr Protoc Essent Lab Tech* 14, 11.1.1–11.1.34 (2017).

[33] Hebditch, M., Carballo-Amador, M. A., Charonis, S., Curtis, R. & Warwicker, J. Protein–Sol: a web tool for predicting protein solubility from sequence. *Bioinformatics* **33**, 3098–3100 (2017). 28575391 10.1093/bioinformatics/btx345 PMC5870856

[34] Geourjon, C. & Deléage, G. SOPMA: significant improvements in protein secondary structure prediction by consensus prediction from multiple alignments. *Bioinformatics* **11**, 681–684 (1995). 10.1093/bioinformatics/11.6.681 8808585

[35] McGuffin, L. J., Bryson, K. & Jones, D. T. The PSIPRED protein structure prediction server. *Bioinformatics* **16**, 404–405 (2000). 10869041 10.1093/bioinformatics/16.4.404

[36] Yang, J. & Zhang, Y. I-TASSER server: new development for protein structure and function predictions. *Nucleic Acids Res.* **43**, W174–W181 (2015). 25883148 10.1093/nar/gkv342 PMC4489253

[37] Heo, L., Park, H., Seok, C. & GalaxyRefine Protein structure refinement driven by side-chain repacking. *Nucleic Acids Res.* **41**, W384–W388 (2013). 23737448 10.1093/nar/gkt458 PMC3692086

[38] Sharma, C., Nigam, A. & Singh, R. Computational-approach Understanding the structure-function prophecy of fibrinolytic protease RFEA1 from Bacillus cereus RSA1. *PeerJ* **9**, e11570 (2021). 34141495 10.7717/peerj.11570 PMC8183432

[39] Colovos, C. & Yeates, T. O. Verification of protein structures: patterns of nonbonded atomic interactions. *Protein Sci.* **2**, 1511–1519 (1993). 8401235 10.1002/pro.5560020916 PMC2142462

[40] Wiederstein, M. & Sippl, M. J. ProSA-web: interactive web service for the recognition of errors in three-dimensional structures of proteins. *Nucleic Acids Res.* **35**, W407–W410 (2007). 17517781 10.1093/nar/gkm290 PMC1933241

[41] Ponomarenko, J. et al. ElliPro: a new structure-based tool for the prediction of antibody epitopes. *BMC Bioinform.* **9**, 1–8 (2008). 10.1186/1471-2105-9-514 PMC2607291 19055730

[42] Rose, P. W. et al. The RCSB protein data bank: integrative view of protein, gene and 3D structural information. *Nucleic Acids Res.* **45**, D271–D281 (2017). 27794042 10.1093/nar/gkw1000 PMC5210513

[43] Kozakov, D. et al. The cluspro web server for protein–protein Docking. *Nat. Protoc.* **12**, 255–278 (2017). 28079879 10.1038/nprot.2016.169 PMC5540229

[44] Yuan, S., Chan, H. C. S. & Hu, Z. Using PyMOL as a platform for computational drug design. *Wiley Interdiscip Rev. Comput. Mol. Sci.* **7**, e1298 (2017).

[45] Abraham, M. J. et al. High performance molecular simulations through multi-level parallelism from laptops to supercomputers. *SoftwareX 1*. **GROMACS**, 19–25 (2015).

[46] Zoete, V., Cuendet, M. A., Grosdidier, A., Michielin, O. & SwissParam A fast force field generation tool for small organic molecules. *J. Comput. Chem.* **32**, 2359–2368 (2011). 21541964 10.1002/jcc.21816

[47] Ke, Q., Gong, X., Liao, S., Duan, C. & Li, L. Effects of thermostats/barostats on physical properties of liquids by molecular dynamics simulations. *J. Mol. Liq*. **365**, 120116 (2022).

[48] Manocha, N. et al. Immunoinformatic approach to design T cell epitope-based chimeric vaccine targeting multiple serotypes of dengue virus. *J. Biomol. Struct. Dyn.* **1–19**10.1080/07391102.2024.2428828 (2024). 10.1080/07391102.2024.2428828 39610038

[49] Rahman, M. N. et al. Immunoselective progression of a multi-epitope-based subunit vaccine candidate to convey protection against the parasite *Onchocerca lupi*. *Inf. Med. Unlocked*. **38**, 101209 (2023).

[50] Madeira, F. et al. The EMBL-EBI job dispatcher sequence analysis tools framework in 2024. *Nucleic Acids Res* gkae241 (2024). 10.1093/nar/gkae241 PMC11223882 38597606

[51] Goulet, D. R. et al. Codon optimization using a recurrent neural network. *J. Comput. Biol.* **30**, 70–81 (2023). 35727687 10.1089/cmb.2021.0458

[52] Goller, C. C., Srougi, M. C., Chen, S. H., Schenkman, L. R. & Kelly, R. M. Integrating bioinformatics tools into Inquiry-Based molecular biology laboratory education modules. *Front. Educ. (Lausanne)*. **6**, 711403 (2021). 35036827 10.3389/feduc.2021.711403 PMC8758113

[53] Donahoe, S. L., Lindsay, S. A., Krockenberger, M., Phalen, D. & Šlapeta, J. A review of neosporosis and pathologic findings of *Neospora Caninum* infection in wildlife. *Int. J. Parasitol. Parasites Wildl.* **4**, 216–238 (2015). 25973393 10.1016/j.ijppaw.2015.04.002 PMC4427759

[54] Dubey, J. P. et al. Gray Wolf (Canis lupus) is a natural definitive host for *Neospora Caninum*. *Vet. Parasitol.* **181**, 382–387 (2011). 21640485 10.1016/j.vetpar.2011.05.018

[55] Dubey, J. P. Review of *Neospora Caninum* and neosporosis in animals. *Korean J. Parasitol.* **41**, 1 (2003). 12666725 10.3347/kjp.2003.41.1.1 PMC2717477

[56] Khazaei-poul, Y., Farhadi, S., Ghani, S., Ahmadizad, S. A. & Ranjbari, J. Monocyclic peptides: Types, synthesis and applications. *Curr. Pharm. Biotechnol.* **22**, 123–135 (2020). 10.2174/1573412916666200120155104 31987019

[57] Ahmed, N. et al. Designing a multi-epitope subunit vaccine against *Toxoplasma gondii* through reverse vaccinology approach. *Mol Biochem. Parasitol* **260**, (2024). 10.1016/j.molbiopara.2024.111655 39521441

[58] Naveed, M. et al. Design of a novel multiple epitope-based vaccine: an immunoinformatics approach to combat SARS-CoV-2 strains. *J. Infect. Public. Health*. **14**, 938–946 (2021). 34119848 10.1016/j.jiph.2021.04.010 PMC8093003

[59] Mohammed, A. A. et al. Epitope-Based Peptide Vaccine against Glycoprotein G of Nipah Henipavirus Using Immunoinformatics Approaches. *J Immunol Res* 2567957 (2020). (2020). 10.1155/2020/2567957 PMC7193299 32377531

[60] Zheng, J. et al. In Silico analysis of Epitope-Based vaccine candidates against hepatitis B virus polymerase protein. *Viruses 2017*. **9**, 112 (2017). 10.3390/v9050112 PMC5454424 28509875

[61] ST, I. et al. In Silico prediction of peptide based vaccine against fowlpox virus (FPV). *Immunome Res* **14**, (2018).

[62] Mashrur, M. N. et al. Development of a novel multi-epitope vaccine candidate for Marburg virus applying advanced immunoinformatics strategies: A computational analysis. *Vacunas* **500461** (2025).

[63] Barua, H. et al. Vacunas developing a novel multi-epitope subunit vaccine to combat Monkeypox virus through an immunoinformatics approach. (2025). 10.1016/j.vacun.2025.500490

[64] Innes, E. A., Andrianarivo, A. G., Björkman, C. & Williams, D. J. L. Conrad, P. A. Immune responses to *Neospora Caninum* and prospects for vaccination. *Trends Parasitol.* **18**, 497–504 (2002). 12473366 10.1016/s1471-4922(02)02372-3

[65] Xagorari, A. & Chlichlia, K. Toll-like receptors and viruses: induction of innate antiviral immune responses. *Open. Microbiol. J.* **2**, 49–59 (2008). 19088911 10.2174/1874285800802010049 PMC2593046

[66] Ayazian Mavi, S. et al. Assessment of the immunogenicity and protective efficiency of a novel dual-promoter DNA vaccine, harboring SAG1 and GRA7 genes, from RH strain of *Toxoplasma gondii* in BALB/c mice. *Infect. Drug Resist.* **12**, 2519–2530 (2019). 31616167 10.2147/IDR.S209270 PMC6699518

[67] Naveed, M. et al. A vaccine construction against COVID-19-Associated mucormycosis contrived with Immunoinformatics-Based scavenging of potential Mucoralean epitopes. *Vaccines (Basel)* **10**, (2022). 10.3390/vaccines10050664 PMC9147184 35632420

