# Supplementary material for: In silico design of novel multi-epitope peptide vaccine against Neospora caninum induced cattle abortion targeting extracellular GRA2 and Nc-p43 protein
Source: Sci Rep. 2025 Nov 26;15:42169. doi: 10.1038/s41598-025-26139-1 (PMC12658060; doi:10.1038/s41598-025-26139-1)
Supplement: Supplementary file 1 — Supplementary Material 1 [file 41598_2025_26139_MOESM1_ESM.pdf]

Supplementary File

Supplementary Table S1. Antigenicity, transmembrane topology, and physicochemical properties of the selected two proteins.

Characteristics	Dense Granule Protein 2	Surface Protein NC-P43
Antigenicity (>0.4)	0.6605	0.8216
Topology	Outside	Outside
Number of amino acids	193	400
Theoretical isoelectric point (pI)	5.05	5.49
Molecular Weight	20589.82	42051.07
Formula	$C_{876}H_{1425}N_{269}O_{296}S_4$	$C_{1827}H_{2876}N_{510}O_{593}S_{18}$
Total Number of atoms	2870	5824
(Asp + Glu)	29	45
(Arg + Lys)	23	37
Half-life	30 hours (mammalian reticulocytes, in vitro). >20 hours (yeast, in vivo). >10 hours (Escherichia coli, in vivo).	30 hours (mammalian reticulocytes, in vitro). >20 hours (yeast, in vivo). >10 hours (Escherichia coli, in vivo).
Instability Index	68.28	48.70
Aliphatic Index	71.81	70.25
GRAVY	-0.493	-0.293

Supplementary Table S2. Antigenicity, allergenicity, toxicity, and topology of the two chosen epitopes on B cells. Selected Epitope (Green color)

**Proteins Name**	**Epitope Sequence**	**Antigenicity (>0.4)**	**Allergenicity (Non-Allergen)**	**Topology (Outside)**	**Toxicity (Non-Toxin)**
**Dense Granule Protein 2**	RRRSGAPREGGENENGG	0.8587	ALLERGEN	Inside	Non-Toxin
	GGENENG	2.5551	ALLERGEN	Outside	Non-Toxin
	RGYTSYG	1.5213	NON-ALLERGEN	Inside	Non-Toxin
	NNRTLARRRRA	0.6013	NON-ALLERGEN	Outside	Non-Toxin
	VAKRQQAR	0.6796	NON-ALLERGEN	Inside	Non-Toxin
	ADPVESV	0.9623	NON-ALLERGEN	Outside	Non-Toxin
	QPSSVT	1.1836	ALLERGEN	Inside	Non-Toxin
	EGGENEN	2.1377	ALLERGEN	Outside	Non-Toxin
	ENENGGE	1.6272	ALLERGEN	Outside	Non-Toxin
**Surface Protein NC-P43**	VSGAPFKSENEK	1.1234	NON-ALLERGEN	Outside	Non-Toxin
	EHYPAT	1.1458	NON-ALLERGEN	Inside	Non-Toxin
	GSNPGGG	1.7152	NON-ALLERGEN	Outside	Non-Toxin
	GAGSNPG	1.2053	NON-ALLERGEN	Outside	Non-Toxin
	SDGEDECE	2.4761	ALLERGEN	Outside	Non-Toxin
	PDEHYPAT	1.5295	NON-ALLERGEN	Outside	Non-Toxin
	GGSQPDQ	2.3511	NON-ALLERGEN	Outside	Non-Toxin
	GSNPGGG	1.7152	NON-ALLERGEN	Outside	Non-Toxin
	WVALVYDSQ	2.2408	NON-ALLERGEN	Outside	Non-Toxin
	GLIVCN	3.6170	ALLERGEN	Inside	Non-Toxin
	ESDGEDE	1.9755	ALLERGEN	Outside	Non-Toxin
	GGENGDS	2.9234	ALLERGEN	Outside	Non-Toxin

Supplementary Table S3. Prediction and characterization of CTL epitopes from Dense Granule Protein 2 based on antigenicity, allergenicity, toxicity, and topology

	Epitope	Antigenic score	Topology	Allergenecity	Toxicity
**CTL Epitope for Dense Granule Protein 2**	GGENENGGE	2.0996	Outside	NA	NT
	GENENGGEE	1.8599	Outside	NA	NT
	EVETDVQPS	1.6864	Outside	NA	NT
	VETDVQPSS	1.6536	Outside	NA	NT
	AEVETDVQP	1.6299	Outside	NA	NT
	AEVETDVQPS	1.6063	Outside	NA	NT
	AEVETDVQPS	1.6063	Outside	NA	NT
	GNAEAEEEA	1.5704	Outside	NA	NT
	AAEVETDVQP	1.5168	Outside	NA	NT
	AAEVETDVQP	1.5168	Outside	NA	NT
	PSSVTIDTE	1.4015	Outside	NA	NT
	AFMGVPLSS	1.3751	Outside	NA	NT
	SSELAGSRD	1.3169	Outside	NA	NT
	EAAEVETDV	1.3153	Outside	NA	NT
	EEEAAEVETD	1.2953	Outside	NA	NT

Supplementary Table S4. Immunoinformatic profiling of HTL epitopes from Dense Granule Protein 2 including cytokine induction potential

	**Epitope**	**Antigenic score**	**Topology**	**Allergenecity**	**Toxicity**	**IL4**	**IL10**	**IFN**
**HTL Epitope for Dense Granule Protein 2**	EEEAAEVETDVQPSS	1.4460	Outside	NA	NT	Inducer	Non-inducer	Non-inducer
	VETDVQPSSVTIDTE	1.4202	Outside	NA	NT	Inducer	Non-inducer	Non-inducer
	AEAEEEAAEVETDVQ	1.3897	Outside	NA	NT	Inducer	Non-inducer	Inducer
	NAEAEEEAAEVETDV	1.3807	Outside	NA	NT	Inducer	Non-inducer	Inducer
	EVETDVQPSSVTIDT	1.3788	Outside	NA	NT	Inducer	Non-inducer	Non-inducer
	GNAEAEEEAAEVETD	1.3742	Outside	NA	NT	Inducer	Non-inducer	Inducer
	EAEEEAAEVETDVQP	1.3082	Outside	NA	NT	Inducer	Non-inducer	Inducer
	AEVETDVQPSSVTID	1.3030	Outside	NA	NT	Inducer	Non-inducer	Non-inducer
	AEEEAAEVETDVQPS	1.2663	Outside	NA	NT	Inducer	Non-inducer	Non-inducer
	ETDVQPSSVTIDTEE	1.2522	Outside	NA	NT	Inducer	Non-inducer	Non-inducer

Supplementary Table S5. Immunological assessment of CTL epitopes derived from Surface Protein NC-P43

	Epitope	Antigenic score	Topology	Allergenicity	Toxicity
**CTL Epitope for Surface Protein NC-P43**	GCTGHPDDK	3.0160	Outside	NA	NT
	GETGGENGD	2.8645	Outside	NA	NT
	LGCTGHPDDK	2.8389	Outside	NA	NT
	LGCTGHPDD	2.7970	Outside	NA	NT
	ETGGENGDS	2.2439	Outside	NA	NT
	WVALVYDSQ	2.2408	Outside	NA	NT
	RPYSAVFPG	2.2286	Outside	NA	NT
	TGGENGDSP	2.0521	Outside	NA	NT
	DDGLIVCNE	1.9652	Outside	NA	NT
	SEDDGLIVC	1.9440	outside	NA	NT
	DKGETGGEN	1.9376	Outside	NA	NT
	YLGCTGHPD	1.9073	Outside	NA	NT
	KPAGAGSNP	1.7944	Outside	NA	NT
	TGGENGDSPV	1.7190	Outside	NA	NT

Supplementary Table S6. Comprehensive evaluation of HTL epitopes from Surface Protein NC-P43 for cytokine induction and immunogenicity

	Epitope	Antigenic score	Topology	Allergenicity	Toxicity	IL4	IL10	IFN
**HTL Epitope for Surface Protein NC-P43**	GLIVCNESDGEDECE	1.9834	Outside	NA	NT	Inducer	Non-inducer	Inducer
	DDGLIVCNESDGEDE	1.6908	Outside	NA	NT	Inducer	Non-inducer	Inducer
	EDDGLIVCNESDGED	1.6644	Outside	NA	NT	Inducer	Non-inducer	Inducer
	ESEVIGQVAHCAYSS	1.5734	Outside	NA	NT	Inducer	Non-inducer	Non-inducer
	SEDDGLIVCNESDGE	1.5634	Outside	NA	NT	Inducer	Non-inducer	Inducer
	FSSSFWTGEASGVAG	1.5590	Outside	NA	NT	Non-inducer	Non-inducer	Non-inducer
	KGETGGENGDSPVLR	1.4350	Outside	NA	NT	Non-inducer	Inducer	Non-inducer
	GPDGKAFPDDYMNHH	1.3952	Outside	NA	NT	Non-inducer	Non-inducer	Non-inducer
	AKPAGAGSNPGGGSQ	1.3727	Outside	NA	NT	Non-inducer	Non-inducer	Non-inducer
	NADQWVALVYDSQHS	1.3503	Outside	NA	NT	Non-inducer	Inducer	Non-inducer
	KPAGAGSNPGGGSQP	1.3475	Outside	NA	NT	Non-inducer	Non-inducer	Non-inducer
	KGKPPTGGSRGTTTG	1.3394	Outside	NA	NT	Non-inducer	Inducer	Non-inducer
	LLNVYVQSRESEVIG	1.3365	Outside	NA	NT	Non-inducer	Inducer	Non-inducer

(Dense Granule Protein 2)

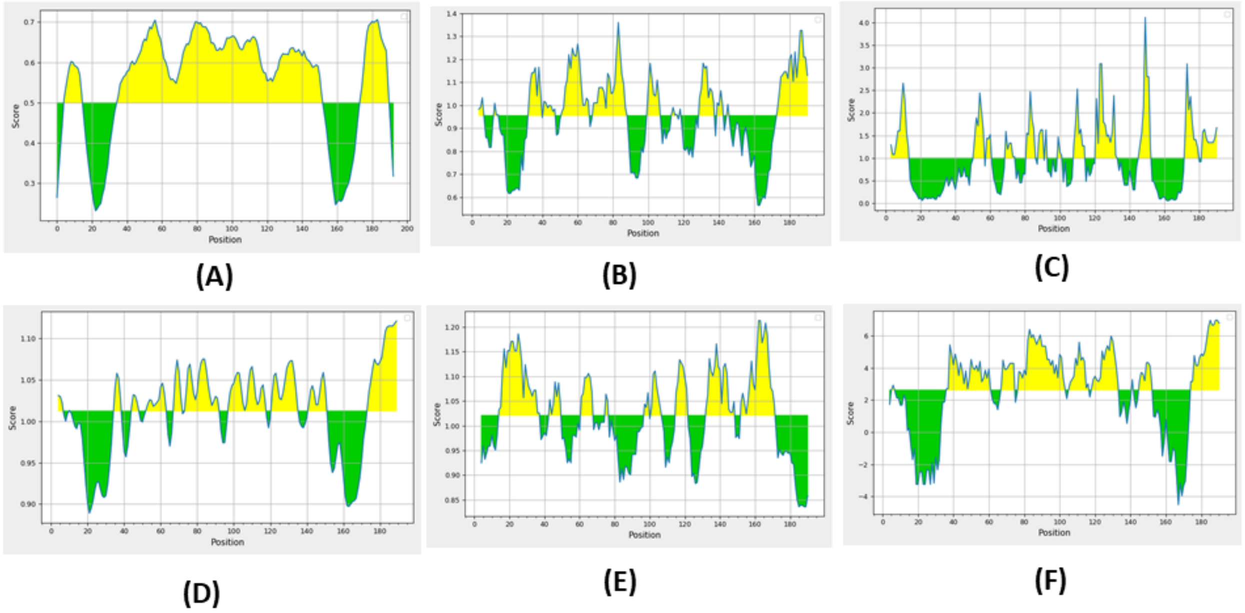

(Surface Protein Nc-p43)

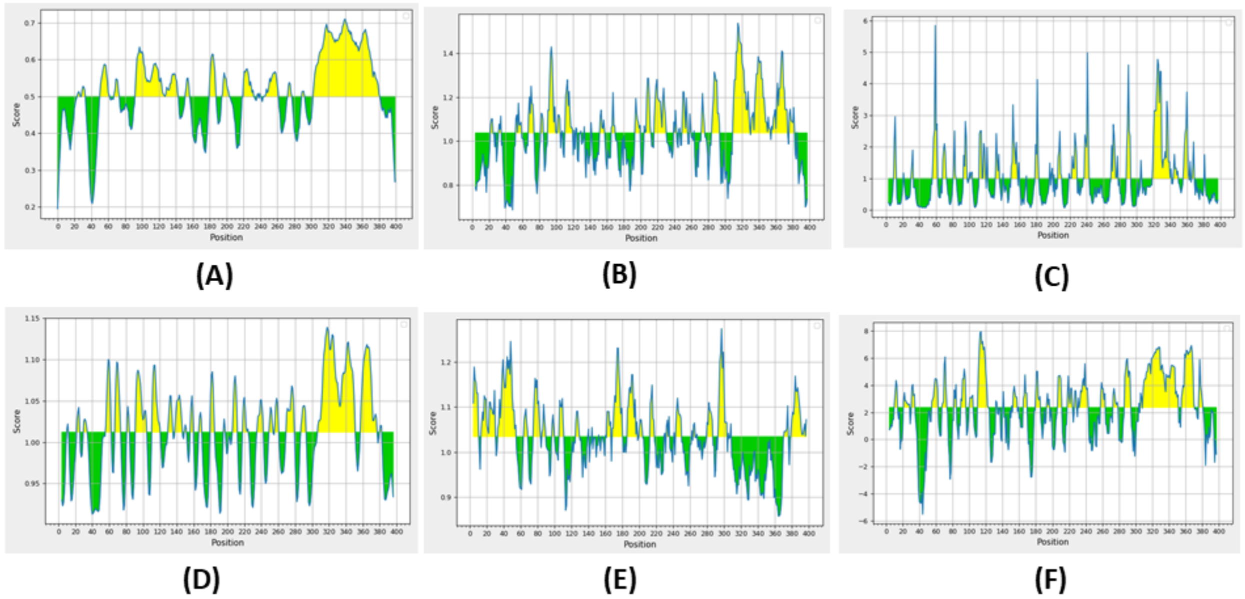

Supplementary Figure S1. The B cell epitopes of the two proteins were predicted utilizing IEDB algorithms based on six methodologies include: (A) Bepipred Linear Epitope Prediction 2.0; (B) Chou & Fasman Beta-Turn Prediction; (C) Emini Surface Accessibility Prediction; (D) Karplus & Schulz Flexibility Prediction; (E) Kolaskar & Tongaonkar Antigenicity; (F) Parker Hydrophilicity Prediction
